# Trends and outcomes in primary health care expenditures in low-income and middle-income countries, 2000–2017

**DOI:** 10.1136/bmjgh-2021-005798

**Published:** 2021-08-13

**Authors:** Matthew T Schneider, Angela Y Chang, Sawyer W Crosby, Stephen Gloyd, Anton C Harle, Stephen Lim, Rafael Lozano, Angela E Micah, Golsum Tsakalos, Yanfang Su, Christopher J L Murray, Joseph L Dieleman

**Affiliations:** 1 Institute for Health Metrics and Evaluation, Seattle, Washington, USA; 2 Institute for Disease Modeling, Bellevue, Washington, USA; 3 Danish Institute for Advanced Study, Copenhagen, Denmark; 4 Department of Clinical Research, University of Southern Denmark, Odense, Syddanmark, Denmark; 5 Department of Global Health, University of Washington, Seattle, Washington, USA

**Keywords:** health economics

## Abstract

**Introduction:**

As the world responds to COVID-19 and aims for the Sustainable Development Goals, the potential for primary healthcare (PHC) is substantial, although the trends and effectiveness of PHC expenditure are unknown. We estimate PHC expenditure for each low-income and middle-income country between 2000 and 2017 and test which health outputs and outcomes were associated with PHC expenditure.

**Methods:**

We used three data sources to estimate PHC expenditures: recently published health expenditure estimates for each low-income and middle-income country, which were constructed using 1662 country-reported National Health Accounts; proprietary data from IQVIA to estimate expenditure of prescribed pharmaceuticals for PHC; and household surveys and costing estimates to estimate inpatient vaginal delivery expenditures. We employed regression analyses to measure the association between PHC expenditures and 15 health outcomes and intermediate health outputs.

**Results:**

PHC expenditures in low-income and middle-income countries increased between 2000 and 2017, from $41 per capita (95% uncertainty interval $33–$49) to $90 ($73–$105). Expenditures for low-income countries plateaued since 2014 at $17 per capita ($15–$19). As national income increased, the proportion of health expenditures on PHC generally decrease; however, the fraction of PHC expenditures spent via ambulatory care providers grew. Increases in the fraction of health expenditures on PHC was associated with lower maternal mortality rate (p value≤0.001), improved coverage of antenatal care visits (p value≤0.001), measles vaccination (p value≤0.001) and an increase in the Health Access and Quality index (p value≤0.05). PHC expenditure was not systematically associated with all-age mortality, communicable and non-communicable disease (NCD) burden.

**Conclusion:**

PHC expenditures were associated with maternal and child health but were not associated with reduction in health burden for other key causes of disability, such as NCDs. To combat changing disease burdens, policy-makers and health professionals need to adapt primary healthcare to ensure continued impact on emerging health challenges.

**Key questions**

**What is already known?**

- Primary healthcare (PHC) has been touted as a means of reaching the health-related Sustainable Development Goals and Universal Health Coverage. While many factors should be assessed to understand the functionality of a nation’s primary healthcare system, one essential input is expenditures for PHC services. However, to date, there is an incomplete understanding of what nations have been spending on these services and how these expenditures relate to changes in health outcomes and intermediate health outputs.

**What are the new findings?**

- Leveraging the work by Schneider *et al* and additional data sources, this research builds on previous attempts by the WHO and the Organization for Economic Cooperation and Development to estimate PHC expenditures for low-income and middle-income countries (LMICs) between 2000 and 2017. It finds that since 2000, PHC expenditures have increased in every income group; however, only in low-income countries have these funds plateaued in recent years. As of 2017, low-income countries were estimated to have spent the largest share of total health expenditures on PHC, 45.8% (IQR 37.3%–53.3%), while lower middle and upper middle income countries spent 39.6% (IQR 37.6%–42.9%) and 37.7% (IQR 33.9%–39.2%), respectively. Using panel regression analyses, this research found that across LMICs, PHC expenditures were significantly associated with improvements in maternal and child health outcomes and outputs but not with other major causes of disease burden, such as non-communicable diseases.

**Key questions**

**What do the new findings imply?**

- For the first time, policy-makers and researchers have the means to include estimates of PHC expenditures into their decision-making processes. While the current estimates of PHC expenditures can only be taken as proxies of what truly is spent on PHC, these are the closest and most complete set of estimates to date. Indications from this research highlights that PHC expenditures are associated with improvements in maternal and child health across LMICs. However, if PHC systems are to truly be the means of reaching the health-related Sustainable Development Goals, policy-makers need to be sure their health system’s first line of defence can address their nations’ changing disease burden.

## Introduction

A well-functioning primary healthcare (PHC) system has the ability to lower the cost of care, improve health-related outcomes across ages and diseases and lead to more equitable healthcare access.^1–7^ Highlighted in the 1978 Declaration of Alma Ata, health advocates have emphasised the role of PHC systems with the hope of achieving ‘health for all’.^8^ This goal has yet to be reached. However, as stated in the 2018 Declaration of Astana, the desire to provide equitable access to healthcare persists.^4 9–11^

Expenditure on PHC is one of many factors that must be measured to assess the capacity of a PHC system.^12 13^ Yet there have been few attempts at systematically measuring these health expenditures that allow for comparisons within and between countries.^14^ The first was conducted in 2016 and updated in 2019 by the Organization for Economic Co-operation and Development (OECD), which estimated PHC expenditures for 22 OECD member nations, most of which are high-income countries.^15 16^ In 2019, Vande Maele *et al*^17^ analysed 36 low-income and middle-income countries (LMICs) for the most recent years of data and found that PHC expenditures ranged from $15 to $60 per capita, representing 31%–88% of current health expenditures. Please note that moving forward in this research, the use of the term total health expenditures is interchangeable with current health expenditures as gross capital formation is removed wherever possible.^18^ Adapting Vande Maele’s approach, in 2020, WHO published PHC expenditure estimates for 88 countries (including high-income countries) for differing years.^19^ These efforts all used country-reported health expenditures, which rely on the System of Health Account framework of categorising these data into groups of healthcare functions (goods and services) and/or providers. The reliance on these expenditure categories has drawbacks; because these categories are disaggregated to large groupings (such as curative inpatient care and over-the-counter drugs), it is not possible to include or exclude certain types of inpatient or pharmaceutical expenditure within a given definition of PHC. Most importantly, countries reporting National Health Accounts have varying degrees of health expenditure reporting completeness and were not originally designed to track PHC expenditures.^17 20 21^ When countries report less detail, the estimation of PHC expenditure varies dramatically, which limits its reliability.^17^

Due to these limitations, the previous attempts to estimate PHC expenditures have only been able to do so for a relatively small number of LMICs and were thus unable to test statistical relationships between changes in PHC expenditures and health outcomes and intermediate outputs.^15 17 19^ Additionally, wide variation in the completeness of data used for these studies suggests that comparisons across countries or time are fraught with challenges. The limited details in the underlying data used required the researchers to assume that no inpatient deliveries were included in PHC expenditures even though basic emergency obstetric care is often considered part of PHC, and a large proportion of these services are provided in an inpatient setting.^22–24^ Finally, an additional assumption was made, that 80% of all expenditures on medical goods were relevant PHC expenditures because primary data on the fraction of pharmaceutical expenditures was not available.^17 19^

To address these gaps, we estimated annual PHC expenditures for 135 LMICs between 2000 and 2017. Our work leveraged a recently published set of complete, estimated, health expenditures for 195 countries.^20^ This complete set of expenditures, which are based on the same country-reported health expenditures used by the WHO and OECD, allowed us to apply a more precise measurement of PHC expenditures than previous research. We also used additional data sources to add expenditures for inpatient vaginal labour and pharmaceutical expenditures on PHC.^23 25^ We present these estimates over time and tested the association between PHC expenditures and multiple health outcomes and intermediate health outputs, such as mortality rates and coverage of skilled birth attendance and vaccinations.

## Methods

### PHC estimation strategy

Given the work conducted by the OECD, Vande Maele and the WHO to understand and propose the most appropriate set of healthcare functions and providers that should be included within the measurement of PHC expenditures, our work did not set out to redefine this measurement strategy.^15 17–19^ Instead, we attempt to estimate a complete time-series of PHC expenditures that closely aligns to the definition currently used by the WHO with incremental improvements for 135 LMICs as defined by the World Bank’s 2019 country classification.^19 26^

To create this full time-series, we drew on previously produced estimates of expenditures for healthcare functions and providers for 195 countries, from 2000 to 2017.^20^ Healthcare functions are groupings aimed at understanding the purpose of funds spent on healthcare services, such as outpatient and inpatient curative care, long-term care and medical goods.^18^ Healthcare providers are organisations and actors that deliver healthcare services, such as hospitals, providers of ambulatory care and providers of preventative care.^18^ Schneider *et al* collected and synthesised all publicly available National Health Accounts reporting healthcare function or provider expenditures.^20^ They estimated healthcare function and provider category combinations for all countries between 2000 and 2017 using these data and a Bayesian statistical model. All estimates include uncertainty intervals, which were calculated using the 2.5th and 97.5th percentile of 1000 estimated samples for each country–year.

As the healthcare function categories do not differentiate between PHC and non-PHC medical goods, we used data from IQVIA Analytics Link to estimate the proportion of health expenditures on prescription pharmaceuticals that we considered part of PHC for all countries and years in our study.^27 28^ To identify the proportion of prescribed pharmaceutical expenditures for PHC within the IQVIA data, the molecule name was matched with the WHO Essential Medicine List.^29^ We applied these country–year estimates of the proportion of prescribed pharmaceuticals that are for PHC to the most appropriate cross-classification of healthcare function and provider expenditure estimates. Additional descriptive and methodological details are in online supplemental appendix A.

10.1136/bmjgh-2021-005798.supp1 Supplementary data

Inpatient vaginal delivery is often considered part of PHC expenditure, but these specific expenditures are not clearly identified in National Health Account data and are excluded from existing OECD and WHO methods to estimate PHC expenditure.^25^ To incorporate inpatient vaginal delivery expenditures in our estimates of PHC, we used survey, administrative and literature sources to estimate country-specific volume and cost of inpatient vaginal delivery for all countries between 2000 and 2017.^30–43^ Additional details of the sources and methods used are provided in online supplemental appendix B.

As presented in table 1, we combined the estimates of PHC-related prescribed pharmaceuticals and inpatient vaginal delivery expenditures with the healthcare function and provider expenditure estimates identified as primary healthcare from previous research to create our estimate of PHC expenditures.^15 17^ Given the granularity of healthcare function and provider expenditure estimates, we were able to include more granular definitions than previous research and make estimates for all LMICs. Specifically, in addition to estimates of PHC-related prescribed medicines and inpatient vaginal deliveries, we included general outpatient, dental, home-based and other outpatient curative care, long-term outpatient and home-based care, preventive care, over-the-counter drugs and other medical non-durable goods, as well as a share of health system administration and governance expenditures. Table 1 presents the included categories and compares our approach with the WHO and OECD strategies. Furthermore, because the archetypal definition of PHC are services provided in a PHC setting, such as an ambulatory setting as proposed by the OECD, we additionally focus in this study on the amount of PHC expenditures that are spent within providers of ambulatory care (healthcare provider categories HP3).^12 15 16 44^

**Table 1:** Comparison of categories included in different measurements of primary healthcare expenditures

Health expenditure categories	Healthcare functions	This research		Previous research	
		PHC	PHC ambulatory	WHO GHED	OECD 2019
		1	2	3	4
General outpatient curative care	HC 1.3.1	Included	Ambulatory settings	Included	Ambulatory settings
Dental outpatient curative care	HC 1.3.2	Included	Ambulatory settings	Included	Ambulatory settings
Curative outpatient care not elsewhere classified	HC 1.3.nec*	Included	Ambulatory settings	Included	Excluded
Home-based curative care	HC 1.4	Included	Ambulatory settings	Included	Excluded
Long-term outpatient care	HC 3.3	Included	Ambulatory settings	Included	Excluded
Long-term home-based care	HC 3.4	Included	Ambulatory settings	Included	Excluded
Preventive care	HC 6	Included	Ambulatory settings	Included	Included
Prescribed medicines	HC 5.1.1	Country–year estimated proportion based on essential medicines	Country–year estimated proportion based on essential medicines in ambulatory settings	80%	Excluded
Over-the-counter drugs	HC 5.1.2	Included	Ambulatory settings	80%	Excluded
Other medical non-durable goods	HC 5.1.3	Included	Included	80%	Excluded
Therapeutic appliances and other medical goods	HC 5.2	Excluded	Excluded	80%	Excluded
Medical goods not elsewhere classified	HC 5.nec*	Excluded	Excluded	80%	Excluded
Health system admin and governance	HC 7	Share of PHC/total expenditure	Share of PHC in ambulatory setting/total expenditure	80%	Excluded
Inpatient vaginal delivery	NA	Included	Excluded	Excluded	Excluded

Components of various measurements of primary healthcare expenditures. These include two measurements from this research: one from the WHO Global Health Expenditure Database (WHO GHED) and another from the Organization for Economic Cooperation and Development (OECD).

*Created when reported total is greater than sum of subcomponent. Healthcare functions are sourced from the 2011 System of Health Accounts.

HC, healthcare functions; NEC, not elsewhere classified; PHC, primary healthcare.

### PHC trends analysis

Using the PHC expenditure estimates identified in column 1 of table 1, we estimated the relationship between PHC expenditures and gross domestic product per capita. We used a generalised additive model with non-linear penalised splines to estimate how the share of total health expenditures that were for PHC changed as gross domestic product per capita increased after controlling for trends in time. This is in line with previously published studies.^45^ Using a similar approach, we estimated the relationship between income and the composition of PHC expenditures, inside and outside of ambulatory settings and non-PHC expenditures. The generalised additive models used the mgcv package and were run on R V.3.6.0.^46^

### PHC outcome analysis

We measured the association between PHC expenditures as share of total health expenditures and health outcomes and intermediate health outputs of interest using multivariate panel regression models (equation 1). The set of outcomes and outputs used as dependent variables ($y_{it}$) were those that had previously been identified as theoretically associated with PHC services and had available national estimates for all LMICs during the study period.^13 14^ The health outcomes used as dependent variables were: (1) age-standardised mortality rates, (2) maternal mortality rate, (3) neonatal mortality rate, (4) children under 5 years old mortality rate, (5) non-communicable disease disability-adjusted life years, (6) communicable disease disability-adjusted life years and (7) prevalence of diabetes. The intermediate health outputs used were: (1) coverage of the fourth antenatal care visit (ANC4), (2) coverage of the third dose of diphtheria-tetanus-pertussis vaccine, (3) measles vaccination coverage, (4) smoking prevalence of people of reproductive age, (5) antiretroviral treatment coverage, (6) health worker density, (7) coverage of births with a skilled birth attendant present, (8) Universal Health Coverage (UHC) effective coverage index and (9) the Healthcare Access and Quality Index. All dependent variables were from the Global Burden of Disease.^47–50^ A more linear relationship was found between the independent and dependent variables if those that are measured as a proportion were logit transformed, and the others were natural log transformed. Our variable of interest, PHC expenditures divided by total health expenditures and total health expenditures per capita were included in all models, as were country and year fixed effects that account for unobserved country differences and global time trends. The covariates ($X_{it}$) considered for inclusion were initially determined based on availability across countries and years, as well as their established importance with the health outcomes and outputs of interest. Then using a stepwise process, we identified the most parsimonious set of country and time varying covariates for each dependent variable. These consisted of average years of education, total fertility rate, prevalence of HIV, the number of hospital beds, lag distributed income, urbanicity, proportion of population over 65 years old and the share of health expenditures that government and out-of-pocket makes up.^47^ Country and time are represented in equation 1 as i and t, respectively.

$y_{it}=\alpha +\gamma_{i}+\gamma_{t}+\beta_{1}{\frac{PHCExpenditures}{TotalHealthExpenditures}}_{it}+\beta_{2}Totalhealthexpenditures_{it}+\beta_{3}X_{it}+\varepsilon_{it}$

Using equation 1, when the dependent variable is log transformed, $\beta_{1}$—the coefficient of our variable of interest—can be interpreted as a 1% change in PHC expenditures as a share of total health expenditures relates to a exp($\beta_{1}$)−1 change in the dependent ($y_{it}$) variable. The coefficients’ SEs were Huber-White robust adjusted to address heteroskedasticity and are clustered by years to address any serial correlation over time. In addition, since the relationship between PHC expenditures and 16 health outcomes and outputs are analysed, we adjust for multiple hypotheses using Bonferroni correction.^51–54^ The panel regressions were conducted using the plm package in R V.3.6.0.^55^ Additional sensitivity analyses are presented in the online supplemental appendix E, including binary analyses and random effects models for each dependent variable.

### Patient and public involvement

As this study relied on secondary data, patients and the public were not involved in the formulation of this research.

## Results

**Figure 1:**
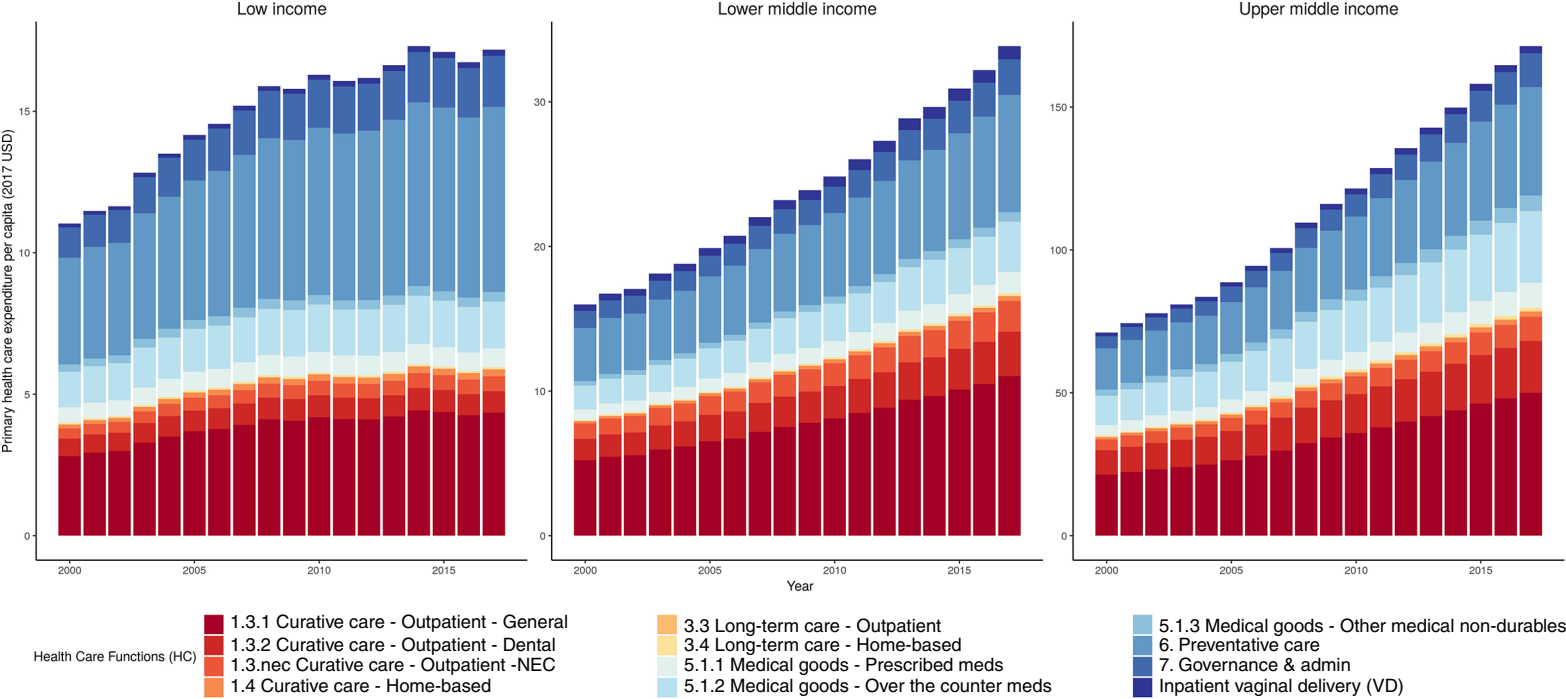
Primary healthcare expenditures per capita estimates by World Bank income groups, 2000–2017. Presents the primary healthcare expenditures, in 2017 US dollars per capita, by healthcare functions and World Bank income groups between 2000 and 2017. Healthcare functions are based on the 2011 system of health account categories with the addition of inpatient vaginal delivery. Vertical axes change between figures for different income groups. NEC, not elsewhere classified.

Figure 1 presents our estimates of PHC expenditures per capita in LMICs between 2000 and 2017. The estimates show that low-income countries have increased from $11 per capita (95% UIs $10 to $12) in 2000 to $17 per capita (95% UIs $15 to $19) in 2017; equating to a 2.64% (95% UIs 2.37% to 2.92%) annual rate of increase. However, the estimates suggest that low-income countries’ expenditure on PHC peaked at $17 per capita ($15–$19) in 2014 and have since plateaued. Meanwhile, lower-middle- and upper-middle-income countries have not shown a similar plateau. Lower-middle income countries were estimated to have spent $16 per capita ($12–$21) in 2000 and $34 per capita ($24–$44) in 2017. We find that in upper-middle-income countries, PHC expenditures totalled $71 per capita ($57–$84) in 2000 and $171 per capita ($139–$202) in 2017. Table 2 presents country estimates of PHC expenditures as both a share of total health expenditures and in US dollars per capita for 2017 using our proposed measurement strategies for PHC (table 1, column 1, ‘PHC’).

**Table 2:** Primary healthcare expenditures with uncertainty intervals in 2017 for all countries

Country	PHC per capita (2017 US$)	PHC as a share of THE
Afghanistan	$26 (17–36)	41.5% (26.9–58.3)
Albania	$119 (77–164)	36.3% (23.5–49.8)
Algeria	$103 (77–125)	38.3% (28.8–46.6)
American Samoa	$247 (185–303)	37.8% (28.2–46.3)
Angola	$46 (34–56)	38.3% (28.8–47.2)
Argentina	$469 (352–575)	37.8% (28.4–46.4)
Armenia	$207 (149–259)	53.3% (38.4–66.9)
Azerbaijan	$103 (77–127)	37.8% (28.3–46.5)
Bangladesh	$16 (10–23)	41.2% (25.0–58.7)
Belarus	$133 (102–161)	38.9% (29.7–47.0)
Belize	$110 (83–136)	39.2% (29.6–48.2)
Benin	$14 (8–20)	39.6% (23.7–56.8)
Bhutan	$25 (13–37)	32.1% (16.1–47.0)
Bolivia	$58 (19–84)	27.7% (9.1–39.9)
Bosnia and Herzegovina	$176 (96–265)	34.6% (18.9–52.1)
Botswana	$208 (125–305)	46.4% (27.8–68.1)
Brazil	$315 (115–486)	34.5% (12.6–53.3)
Bulgaria	$237 (136–337)	35.3% (20.3–50.2)
Burkina Faso	$21 (17–27)	54.5% (42.6–68.0)
Burundi	$16 (12–21)	55.6% (39.9–71.9)
Cambodia	$33 (15–59)	41.8% (19.2–74.0)
Cameroon	$23 (17–30)	39.4% (29.1–51.3)
Cape Verde	$62 (39–85)	38.6% (24.2–53.4)
Central African Republic	$8 (6–10)	37.4% (28.2–47.0)
Chad	$11 (8–13)	38.1% (28.5–46.8)
China	$144 (110–180)	32.7% (25.2–41.0)
Colombia	$173 (129–211)	38.2% (28.6–46.8)
Comoros	$22 (4–35)	32.4% (5.1–50.4)
Congo (Brazzaville)	$22 (14–32)	44.9% (27.4–64.1)
Costa Rica	$322 (249–400)	33.9% (26.3–42.2)
Cuba	$457 (346–553)	39.0% (29.5–47.2)
Côte d'Ivoire	$34 (22–47)	45.5% (30.3–64.0)
DR Congo	$9 (7–10)	53.1% (43.2–63.3)
Djibouti	$22 (16–27)	37.8% (28.5–46.8)
Dominica	$198 (82–398)	41.3% (17.1–83.2)
Dominican Republic	$95 (52–163)	21.3% (11.7–36.5)
Ecuador	$151 (63–245)	29.0% (12.1–47.1)
Egypt	$50 (33–71)	39.3% (25.7–55.5)
El Salvador	$109 (51–179)	34.9% (16.4–57.4)
Equatorial Guinea	$110 (85–133)	40.7% (31.3–49.4)
Eritrea	$11 (8–13)	38.4% (29.0–47.4)
eSwatini	$110 (56–172)	38.4% (19.7–59.9)
Ethiopia	$17 (11–22)	57.5% (36.4–76.4)
Federated States of Micronesia	$55 (34–79)	40.4% (25.1–57.7)
Fiji	$80 (54–108)	40.5% (27.4–54.9)
Gabon	$98 (68–139)	37.1% (25.8–52.4)
Georgia	$105 (68–148)	32.6% (21.1–45.9)
Ghana	$29 (18–46)	44.3% (26.9–69.5)
Grenada	$187 (141–231)	38.7% (29.1–47.8)
Guatemala	$93 (41–144)	35.2% (15.4–54.3)
Guinea	$22 (14–29)	57.0% (35.6–73.5)
Guinea-Bissau	$23 (17–28)	37.2% (27.9–46.4)
Guyana	$159 (103–214)	63.1% (40.7–84.6)
Haiti	$23 (16–29)	48.7% (34.8–63.2)
Honduras	$72 (54–88)	38.6% (29.2–47.6)
India	$30 (12–51)	43.3% (16.7–72.7)
Indonesia	$49 (25–68)	40.2% (20.4–56.3)
Iran	$149 (117–182)	32.9% (26.0–40.2)
Iraq	$67 (27–95)	41.0% (16.4–58.0)
Jamaica	$125 (94–154)	39.0% (29.4–48.0)
Jordan	$79 (41–128)	28.1% (14.4–45.3)
Kazakhstan	$102 (78–129)	33.8% (25.8–42.8)
Kenya	$42 (23–62)	49.6% (27.3–72.9)
Kiribati	$85 (48–123)	38.4% (21.8–55.4)
Kyrgyzstan	$32 (22–44)	38.5% (26.2–53.2)
Laos	$24 (13–40)	41.9% (22.3–68.5)
Lebanon	$167 (79–278)	32.6% (15.5–54.3)
Lesotho	$55 (42–66)	39.4% (29.9–47.1)
Liberia	$24 (11–37)	36.5% (16.1–56.0)
Libya	$154 (117–188)	39.1% (29.8–47.6)
Madagascar	$8 (4–16)	35.1% (17.9–71.9)
Malawi	$22 (17–27)	53.5% (41.9–66.1)
Malaysia	$145 (114–170)	37.2% (29.3–43.7)
Maldives	$420 (195–615)	43.5% (20.2–63.7)
Mali	$17 (10–23)	54.3% (32.7–75.2)
Marshall Islands	$154 (115–192)	37.4% (28.0–46.4)
Mauritania	$18 (12–27)	31.3% (20.6–46.3)
Mauritius	$193 (106–291)	32.0% (17.7–48.3)
Mexico	$177 (139–221)	34.9% (27.3–43.6)
Moldova	$96 (53–134)	50.0% (27.7–70.1)
Mongolia	$68 (44–95)	43.7% (28.5–61.3)
Montenegro	$219 (139–303)	33.8% (21.5–46.8)
Morocco	$65 (32–92)	38.9% (19.4–55.0)
Mozambique	$9 (4–16)	31.8% (13.4–56.9)
Myanmar	$26 (15–37)	47.3% (27.7–65.8)
Namibia	$186 (119–262)	36.0% (23.1–50.6)
Nepal	$25 (15–36)	51.6% (31.7–74.2)
Nicaragua	$80 (55–107)	42.6% (29.3–57.0)
Niger	$12 (7–18)	46.3% (27.0–68.7)
Nigeria	$31 (17–48)	46.6% (24.9–72.0)
North Korea	$30 (22–36)	37.7% (28.2–46.5)
North Macedonia	$163 (125–195)	40.3% (31.0–48.3)
Pakistan	$16 (9–24)	36.8% (21.0–56.1)
Palestine	$150 (108–190)	41.4% (29.9–52.4)
Papua New Guinea	$14 (7–24)	26.8% (13.1–45.8)
Paraguay	$107 (63–167)	28.1% (16.5–44.0)
Peru	$129 (98–157)	39.3% (29.7–47.7)
Philippines	$52 (34–74)	37.5% (24.9–53.8)
Romania	$179 (107–252)	32.4% (19.4–45.7)
Russia	$302 (201–382)	52.7% (35.1–66.7)
Rwanda	$24 (12–41)	51.4% (26.4–88.5)
Saint Lucia	$196 (147–241)	38.8% (29.0–47.6)
Saint Vincent and the Grenadines	$125 (94–154)	39.2% (29.4–48.0)
Samoa	$67 (31–114)	30.3% (13.9–51.2)
Senegal	$28 (20–38)	44.3% (30.8–59.3)
Serbia	$171 (75–386)	39.9% (17.4–90.4)
Sierra Leone	$52 (34–66)	76.8% (50.2–97.4)
Solomon Islands	$42 (32–52)	37.7% (28.3–46.5)
Somalia	$3 (2–3)	37.1% (27.8–46.3)
South Africa	$187 (109–275)	35.8% (20.9–52.6)
South Sudan	$15 (9–20)	52.2% (31.7–70.8)
Sri Lanka	$58 (34–92)	35.8% (21.0–56.1)
Sudan	$29 (22–36)	38.6% (28.9–47.3)
Suriname	$126 (79–181)	36.1% (22.7–51.7)
Syria	$17 (13–21)	38.6% (29.0–47.0)
São Tomé and PrÍncipe	$39 (29–48)	37.3% (28.0–46.3)
Tajikistan	$16 (10–25)	27.9% (17.8–42.4)
Tanzania	$25 (17–33)	61.8% (41.0–81.2)
Thailand	$145 (109–178)	58.8% (44.0–71.8)
The Gambia	$19 (9–29)	46.0% (22.1–68.9)
Timor-Leste	$40 (21–56)	56.7% (30.2–79.3)
Togo	$19 (12–27)	48.8% (30.9–68.0)
Tonga	$84 (44–131)	37.9% (19.8–59.1)
Tunisia	$72 (39–115)	28.2% (15.4–45.2)
Turkey	$132 (79–192)	29.3% (17.7–42.6)
Turkmenistan	$200 (149–247)	37.4% (27.8–46.0)
Uganda	$15 (9–23)	36.1% (21.9–53.6)
Ukraine	$51 (37–66)	29.2% (21.5–37.9)
Uzbekistan	$36 (27–44)	37.8% (28.3–46.2)
Vanuatu	$40 (22–60)	42.1% (23.5–62.4)
Venezuela	$39 (29–47)	39.8% (30.3–48.4)
Vietnam	$43 (23–68)	33.4% (17.5–53.2)
Yemen	$15 (11–20)	36.4% (25.2–47.4)
Zambia	$32 (15–50)	42.4% (20.3–67.3)
Zimbabwe	$56 (32–77)	45.9% (26.2–63.6)

THE, Total health expenditures.

**Figure 2:**
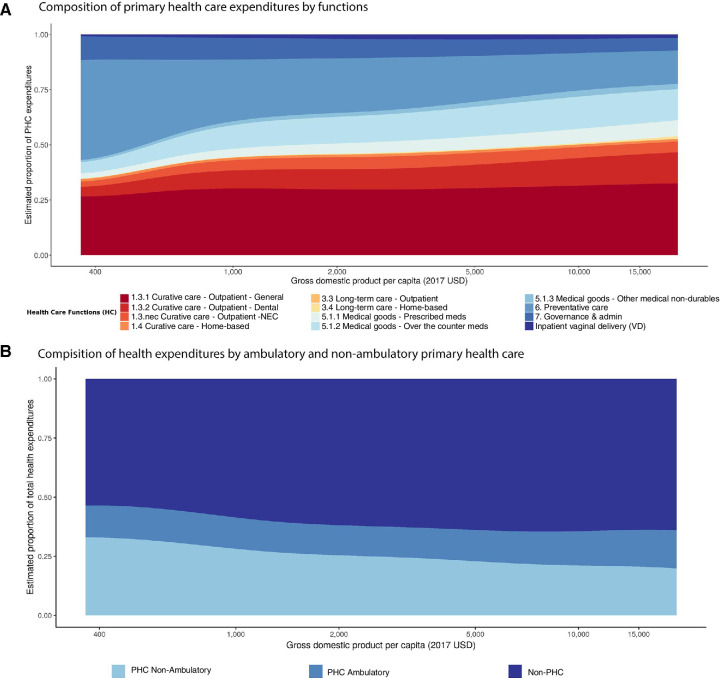
Estimated relationship between primary healthcare expenditures and gross domestic product. Panel A shows the estimated relationship between the components of primary healthcare expenditures and gross domestic product per capita in 2017 US$. Healthcare functions are based on the 2011 system of health account categories. Panel B shows the relationship between gross domestic product per capita in 2017US$ and the composition of total health expenditures by non-primary healthcare, primary healthcare provided by ambulatory care providers and primary healthcare provided by non-ambulatory care providers. NEC, not elsewhere classified.

Figure 2A shows that countries with lower gross domestic product per capita spent more of their PHC expenditures on preventative care, while medical goods and outpatient care made up a large share of PHC expenditures in countries with higher income. Across LMICs, on average 27.6% (min=13.0% and max=42.3%) of prescribed pharmaceutical expenditures were found to be essential medicines and allocated toward PHC. Figure 2B shows the changes in PHC expenditures (inside and outside of an ambulatory setting) and non-PHC expenditures with income. We see that as income increases, PHC expenditures decrease as a share of total health expenditures and that PHC expenditures in an ambulatory setting make up a larger proportion of PHC expenditures.

**Figure 3:**
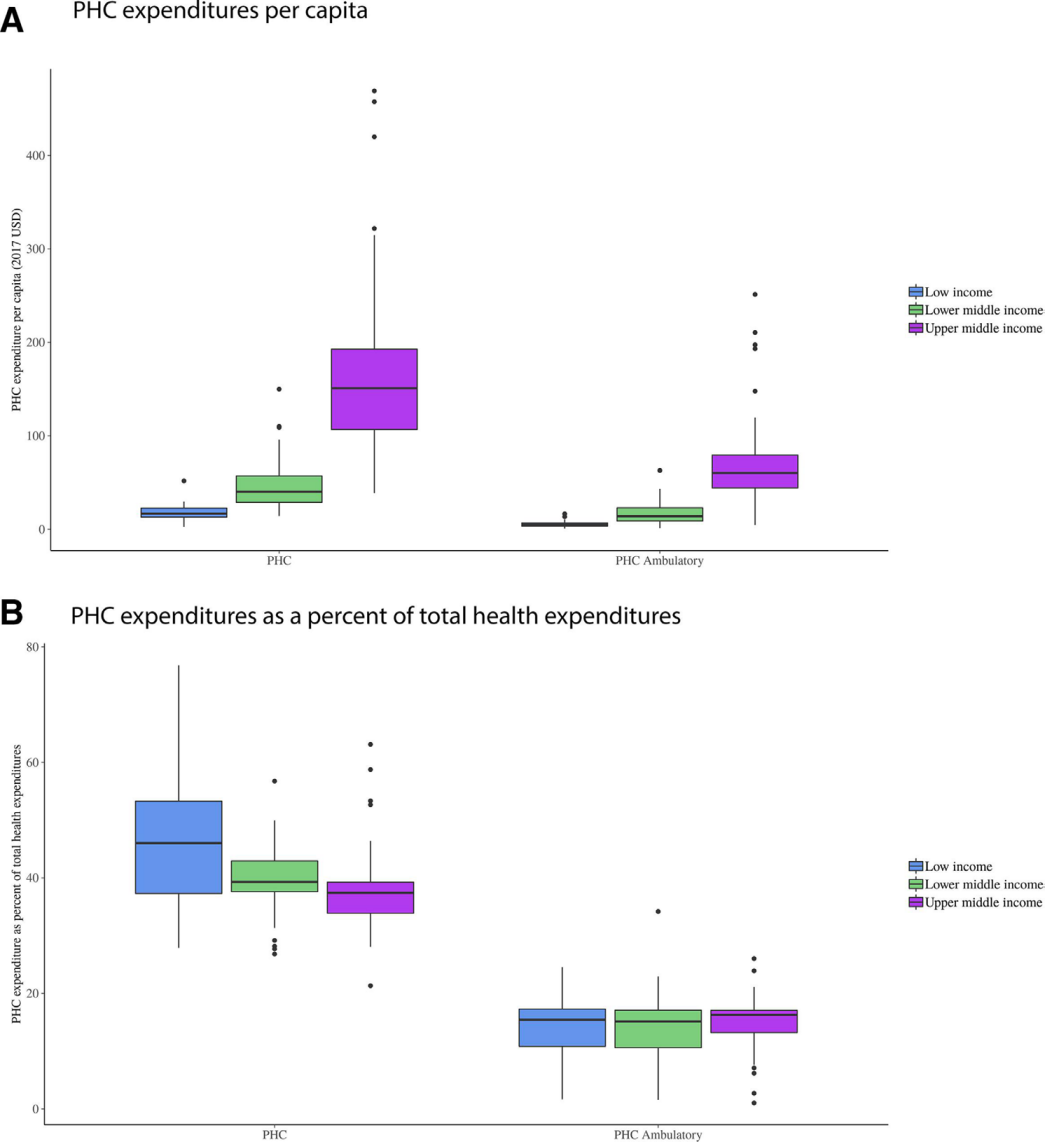
Primary healthcare expenditures by measurement strategies and World Bank income groups in 2017. Panel A presents primary healthcare expenditures per capita (in 2017 US$) by World Bank income groups for two definitions of primary healthcare: (1) across all providers and (2) for only ambulatory providers (as defined within (table 1). Panel B presents primary healthcare expenditures a per cen*t* of total health expenditures by World Bank income groups for the same definitions of primary healthcare outlined above. PHC, primary healthcare.

Figure 3A presents the estimates of PHC expenditures per capita in 2017 US$ across income groups. Overall, PHC expenditures increase with income. For all LMICs, $91.4 per capita (IQR $24.1–$138.5) was estimated to have been spent on PHC. This breaks down into $18.3 (IQR $13.1–$22.7), $47.4 (IQR $28.7–$57.1) and $167.5 (IQR $106.9–$192.7) per capita for low-income, lower middle and upper middle-income countries, respectively. Limiting to ambulatory settings, PHC expenditures drop to $36 (IQR $7–$55) per capita across all LMICs.

When measured as a share of total health expenditures, proportion of PHC expenditure was found to decrease as countries’ income increased (figure 3B). Across LMICs, a median value of 40.2% (IQR 35.9%–42.9%) of total health expenditures was estimated to have been spent on PHC. This breaks down into 45.8% (IQR 37.3%–53.3%), 39.6% (IQR 37.6%–42.9%) and 37.7% (IQR 33.9%–39.2%) for low, lower middle and upper middle income countries, respectively. Conversely, by limiting PHC expenditures to PHC expenditure in only ambulatory settings, we see that the median estimate of PHC expenditures increased slightly as income increased.

**Figure 4:**
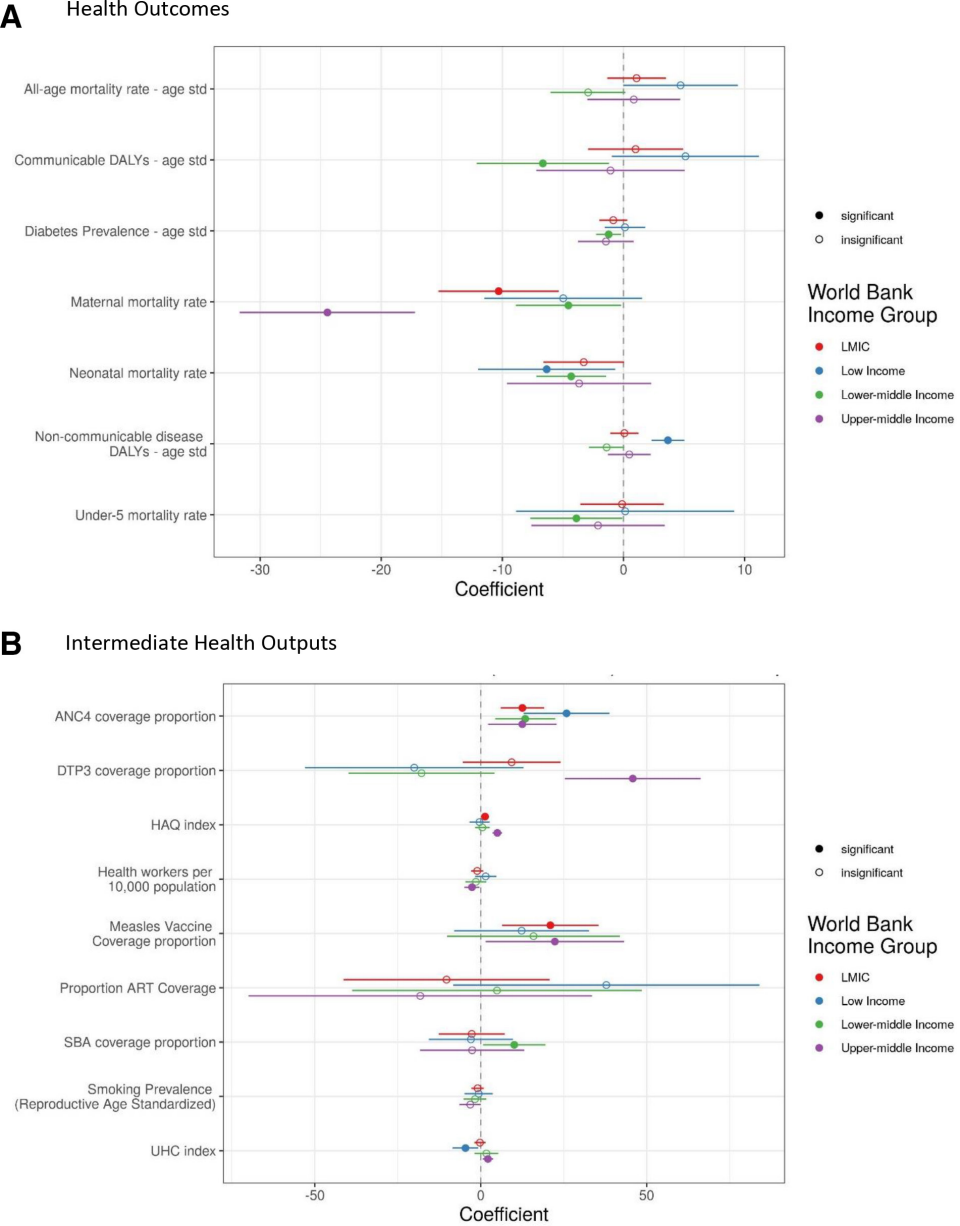
Fixed effect models coefficients of primary healthcare as a share of total health expenditures. Panel A Presents the regression coefficients between primary healthcare as a share of total health expenditures and log-transformed health outcomes. Panel B presents the regression coefficient between primary healthcare as a share of total health expenditures and intermediate health outputs (coverage and proportion outputs are logit-transformed). Seperate regressions (as defined in equation 1) were estimated for each outcome and output using data for countries within World Bank income groups (indicated by the colors). All standard errors (indicated by horizontal bars) are Huber-White and Bonferroni adjusted. Significant findings are p value <0.05 (complete regression outputs are presented in online supplemental appendix D). ANC, antenatal care; ART, antiretroviral therapy; DALYs, disability-adjusted life years; DTP, diphtheria, tetanus and pertussis; HAQ, healthcare access and quality index; LMIC, low-income and middle-income countries; SBA, skilled birth attendance; UHC, Universal Health Coverage effective coverage index.

Figure 4A shows that after adjusting for income, total health expenditures and other social and demographic difference across LMICs, additional PHC expenditures as a share of total health expenditures was associated with lower maternal mortality rate (p value≤0.001). However, when limiting the regression models to specific income group’s data, we found significant differences between groups. Both lower and upper middle income countries were found to have statistically significant improvements in maternal mortality with increased PHC expenditures. Low-income and lower middle income countries were found to have significant improvements in neonatal mortality with additional PHC expenditures. Lower middle income countries with a higher share of PHC expenditures had improvements in communicable burden of disease (p value≤0.01), diabetes prevalence (p value≤0.01) and under-5 mortality. Unexpectedly, low-income countries that spent more on PHC had significantly higher non-communicable disease burden.

Figure 4B shows that across study countries, an increase in PHC expenditures as a share of total health expenditures is related to statistically significant improvements in coverage of four or more antenatal care visits (ANC4) (p value≤0.001), health access and quality index (p value≤0.05) and coverage of measles vaccination (p value≤0.001). Subanalyses, limiting data for only countries within the same income groups, show that all income groups had statistically significant improvements in ANC4 coverage with more of their health expenditures being spent on PHC. We also see upper middle income countries that spent more on PHC showed significant improvements in coverage of the third dose of diphtheria-tetanus-pertussis (p value≤0.001), and of the measles (p value≤0.05) vaccines, and the health access and quality (p value≤0.001) and UHC (p value≤0.01) indices. Lastly, this subanalyses indicates that lower middle incomes countries that spent more on PHC had statistically significant higher coverage of skilled birth attendance (p value≤0.05). However, this analysis also showed that health worker density decreased for upper middle income countries (p value≤0.01) and UHC index decreased in low-income countries (p value≤0.01), which spent more on PHC. Additional sensitivity analyses, including 10-year lagged variables and disaggregating PHC expenditures by ambulatory and non-ambulatory providers are presented in the online supplemental appendix F reinforcing these main findings.

## Discussion

As found in the previous research by Vande Maele and Mueller, there is not one resource originally created to measure PHC expenditures overtime and across countries.^15 17 19^ However, the utility of being able to track PHC expenditures is evident. Our work sought to make incremental improvements to the PHC expenditure measurement strategy presented on the Global Health Expenditure Database at the time of this research, while also estimating a complete time-series of PHC expenditures with uncertainty intervals for 135 LMICs from 2000 to 2017.

We found that PHC expenditures in LMICs increased from $209.6 billion (UIs $169.0–$248.1) in 2000 to $574.2 billion (UIs $469.7–$675.6) in 2017. While middle-income countries have continued to see relatively steady annual increases in PHC expenditures, PHC expenditure in low-income countries has plateaued since 2014 when they spent $17 per capita ($15–$19), which equates to 47.0% (40.8%–52.4%) of total health expenditures in these countries. As PHC was found to make up a relatively similar share of total expenditures over the study period, these findings reflect the overall trend in health expenditures in low-income countries, which grew sharply during the 2000s as the world rallied behind the Millennium Development Goals but has slowed since.^56–59^

PHC expenditure in LMICs was associated with improvements in maternal and child health, but not, in general, more broadly. This supports findings from previous research emphasising the ability of PHC to mediate maternal and child health burden but also emphasised the calls for adapting PHC systems to address more chronic and non-communicable diseases.^60–64^ As the health burden globally and in LMICs moves to be more and more focused on NCDs, it is critical that PHC spending be re-envisioned to ensure that it can have positive health gains for all major health focus areas and ages groups.^65 66^

The use of pharmaceutical costs data and the WHO essential medicines list showed an average of 27.6% of prescribed pharmaceuticals were spent for PHC across LMICs. This is in stark contrast to the 80% assumed by the current definition used by the WHO.^19^ In fact, the maximum estimated proportion of essential prescribed pharmaceuticals was found to be 40.3% and 42.3% in Zimbabwe and Sierra Leone, respectively. Additionally, as with the methods employed by WHO to estimate PHC expenditures, any pharmaceuticals or medical good expenditures captured within other categories of PHC (table 1) would be counted fully.

As proposed originally by Starfield and more recently by Baillieu *et al*, PHC services should ideally be provided within PHC settings (such as a health post or centre) and by primary care providers.^12 44 67^ While the System of Health Accounts framework that this research relies on does not provide refined details of healthcare facility or provider types (such as primary care providers or health posts and clinics), it does allow for the distinction between providers of ambulatory care from hospitals and long-term facilities.^18^ Using this detail, we demonstrated how trends in PHC expenditures within ambulatory settings make up a smaller share of total health expenditures in lower income countries. This distinction may be due to multiple reasons and requires further research; one potential hypothesis for this observation could be due to underlying patient perception that ambulatory settings do not provide the same quality of PHC services as hospitals in lower income countries, thus increasing utilisation of services provided in the hospital setting.^68^ An important dimension that would aid in understanding the related incentives or disincentives to use certain types of care is the source of PHC expenditures. Specific sources, such as government health schemes, private insurers or out of pocket, may drive the types of care that is both sought out and provided in a health system.^69 70^ Country-reported estimates of PHC expenditures within National Health Accounts were compared with our two estimate (table 1 – PHC and PHC ambulatory); interestingly, our estimates of PHC within ambulatory settings more closely aligned with these country-reported estimates (online supplemental appendix C). While additional research is warranted to fully understand this observation, this close alignment could be interpreted as countries actively choosing the define their PHC expenditures within ambulatory settings or they are limited in their ability to breakdown health expenditures, such as within hospitals, and so they choose to use what is available, not what is ideal.

PHC is often considered the backbone of a health system and touted as a means to reach the Sustainable Development Goals. In addition, it is an essential component to reaching the Sustainable Development Goals, such as providing Universal Healthcare Coverage and reducing maternal, neonatal and under-5 mortality.^4 10 13 71^ We explored the relationship between PHC expenditures and health outcomes and intermediate health outputs. The outcomes and outputs used in this research were identified as core indicators that are used to measure PHC performance across countries.^13^ After adjusting for relevant covariates, there was an association between higher PHC expenditure and improvements in maternal mortality rate, ANC4 coverage, measles vaccination coverage and an index measuring access and quality of healthcare. While these findings are very promising, we found heterogeneity between income groups and many insignificant results. Most concerning, we do not find statistically significant associations between PHC expenditures and other indicators of broad health gains, such as all-age adult mortality, communicable and non-communicable disease burden and the UHC effective coverage index. Given the import role that PHC can and should play in improving health for these types of health outcomes, ongoing efforts are needed to understand these null findings, identify positive outliers and address what can be done to ensure that PHC has the broadest impact possible.^62–64^ While these findings are concerning, we emphasise that the use of observational data cannot draw causality between PHC expenditures and health outcomes and outputs. Still these troublesome findings highlight interesting differences in the relationships between income groups. Specifically, future research is needed into the interaction between PHC expenditures and key factors such as policies that drive differences across national health system, patient perception of healthcare quality and provision within countries, availability and quality of services within categories of care and how to ensure that PHC impacts key healthcare areas beyond maternal and child health.

### Limitations

This research has several limitations. Most importantly, as with previous work, this research relies on data and a framework that were not created to track and measure PHC expenditures across countries and time.^15–17 20 21^ As a consequence, these findings and results should be taken as proxy measures of true PHC expenditures. The estimates of health expenditures that were used relied on income to fill in missing data points that may cause blending of the relationships between PHC expenditures and income. For this reason, we do not include a measure of income in our final regression analyses. Still, this may have resulted in removal of what otherwise would be significant findings but should not change those that were found to be significant. Additionally, the underlying estimates of expenditures by healthcare functions and providers relied on more consistent and detailed reporting, often from higher income countries to estimate missing values. While all attempts were made to account for geographic and contextual difference, there is still uncertainty in the estimates that is accounted for in the UIs provided.^20^

Our estimation strategy to understand the proportion of pharmaceutical expenditures that are for PHC relied on sales data from national audits but for a majority of high-income countries and only between 2014 and 2017.^27 28^ It is unclear exactly how the influence of these costs would influence our results; one potential is that low-income settings may have additional PHC pharmaceuticals and fewer expenditures on non-essential medicines, leading to underestimations. Additionally, it is known that country-specific essential medicines lists on average include additional drugs than within the WHO list, leading to a potential underestimation of the proportion of prescribed pharmaceutical spending for PHC.^72^ This is possibly countered by the inclusion of more expensive drugs, such as cancer treatments, included in the essential medicines list that may fall outside the PHC domain. The available cost data for inpatient vaginal delivery is limited to only a few country–years, and thus, the reliance on more robust estimates of cesarean section costs was built into our methods. Ultimately, these estimates are considered a first-order estimation and although they provide a valuable addition, they require additional research to substantiate. The financing source of health expenditures for PHC is an important dimension that this study was not able to incorporate in the current iteration. Lastly, the relationships between PHC expenditures and health outcome and outputs presented here are drawn from estimates that contain uncertainty, but we currently do not incorporate these into the panel regression analysis.

## Conclusion

PHC systems aim to improve access to healthcare services equitably and are a means for countries to progress towards the health related Sustainable Development Goal.^63 67 71 73^ PHC expenditures are a crucial component for assessing the priority placed on a national PHC system. This study found that across LMICs, funding for PHC systems has increased between 2000 and 2017; however, these funds have plateaued in low-income countries and that PHC expenditure was most associated with maternal and child health outcomes, but not health outcomes related to NCDs or adult health. Further research is needed to understand if this trend of flattening PHC expenditures is leading to greater health disparities in low-income countries and how to ensure that PHC expenditures address all major health focus areas, including NCDs and adult health.

## Data Availability

Data are available in a public, open access repository. The data that support the findings of this study will be made publically available at IHME’s Global Health Data Exchange website (http://ghdx.healthdata.org/) on publication.
