# Supplementary material for: Trends and outcomes in primary health care expenditures in low-income and middle-income countries, 2000–2017
Source: BMJ Glob Health. 2021 Aug 13;6(8):e005798. doi: 10.1136/bmjgh-2021-005798 (PMC8362721; doi:10.1136/bmjgh-2021-005798)
Supplement: Supplementary data [file bmjgh-2021-005798supp001.pdf]

Appendix

Contents

Appendix 1
A. Detailed methods for estimating prescribed pharmaceutical expenditures for primary health care 1
B. Detailed methods for estimating inpatient vaginal delivery expenditures 14
C. Comparison of estimates of primary health care expenditures versus country reported primary health care expenditures from national health accounts 21
D. Complete outcome/output fixed effects regression outputs 23
E. Binary relationships between PHC expenditures and outcomes/outputs 31
F. Sensitivity analyses: 10-year difference, Mundlak, and ambulatory split model outputs 39
References 45

A. Detailed methods for estimating prescribed pharmaceutical expenditures for primary health care

We used data from IQVIA Analytics Link to estimate the proportion of health expenditures on prescription pharmaceuticals that we considered part of PHC.[1] IQVIA data obtained for our research contained prescribed pharmaceutical sales records for 49 countries, between 2014 and 2018. These expenditures were reported by the pharmaceutical molecule name as a total for the country and/or by the settings the prescriptions were purchased - hospitals or retailers. To identify the proportion of prescribed pharmaceutical expenditures for PHC within the IQVIA data, the molecule name was matched with the WHO Essential Medicine List.[2] Any pharmaceuticals not matched with the Essential Medicine List were assumed not to be for PHC. Of the 460 pharmaceutical molecules in the WHO Essential Medicine List, 289 were matched within the IQVIA data. A complete list of countries within the IQVIA data and the settings reported, as well as a list of pharmaceutical molecules matched between the WHO Essential Medicine List and IQVIA found below.

We used Spatio-Temporal Gaussian Process Regression (ST-GPR) to estimate the proportion of prescribed pharmaceutical expenditures that are for PHC within hospitals, retailers, and as an aggregate for all countries between 2000 and 2017.[3] We modelled expenditures for prescribed essential pharmaceuticals as a proportion of total prescription pharmaceutical expenditures reported in the IQVIA data, for retail and hospital settings separately and as an aggregate. All covariates considered were either from the Global Burden of Disease study or estimates produced by Schneider et al.[4][Schneider] Covariates considered for the linear first stage of ST-GPR for all models are shown in Table A.4. Covariates for each model were selected based on Akaike information criterion using the base step function in R version 3.6.0 and are presented in the appendix.

We applied these country-year, setting-specific estimates of the proportion of prescribed pharmaceuticals that are for PHC to the most appropriate cross-classification of health care function and provider expenditure estimates. For prescribed pharmaceutical expenditures (HC 5.1.1) within hospitals (HP 1) and ambulatory care providers (HP 3) we

multiplied the country-year specific estimate of prescribed medicines that are for PHC within hospitals. For prescribed pharmaceutical expenditures within retailors of medical goods (HP 5) we applied the country-year specific estimate of prescribed pharmaceutical that are for PHC within retailers. For all other health care providers with any amount of prescribed pharmaceutical expenditures we applied the average of the two settings – hospitals and retailers - for that given country-year.

Table A.1 Channels available by country in IQVIA Analytics Link

Country	Channel
AUSTRALIA	HOSPITAL
AUSTRALIA	RETAIL
AUSTRIA	HOSPITAL
AUSTRIA	RETAIL
BELARUS	HOSPITAL
BELARUS	RETAIL
BELGIUM	HOSPITAL
BELGIUM	RETAIL
BOSNIA-HERZEGOVINA	COMBINED
BULGARIA	HOSPITAL
BULGARIA	RETAIL
CANADA	HOSPITAL
CANADA	RETAIL
CHINA	HOSPITAL
CHINA	RETAIL
CROATIA	HOSPITAL
CROATIA	RETAIL
CZECH REPUBLIC	HOSPITAL
CZECH REPUBLIC	RETAIL
FINLAND	HOSPITAL
FINLAND	RETAIL
FRANCE	HOSPITAL
FRANCE	RETAIL
GERMANY	HOSPITAL
GERMANY	RETAIL
HUNGARY	HOSPITAL
HUNGARY	RETAIL
INDIA	COMBINED
INDONESIA	HOSPITAL
INDONESIA	RETAIL
IRELAND	HOSPITAL
IRELAND	RETAIL
ITALY	HOSPITAL

ITALY	RETAIL
JAPAN	HOSPITAL
JAPAN	RETAIL
KAZAKHSTAN	HOSPITAL
KAZAKHSTAN	RETAIL
LATVIA	HOSPITAL
LATVIA	RETAIL
LITHUANIA	HOSPITAL
LITHUANIA	RETAIL
MALAYSIA	COMBINED
NETHERLANDS	HOSPITAL
NETHERLANDS	RETAIL
NEW ZEALAND	HOSPITAL
NEW ZEALAND	RETAIL
NORWAY	HOSPITAL
NORWAY	RETAIL
PHILIPPINES	HOSPITAL
PHILIPPINES	RETAIL
POLAND	HOSPITAL
POLAND	RETAIL
PORTUGAL	HOSPITAL
PORTUGAL	RETAIL
PUERTO RICO	HOSPITAL
PUERTO RICO	RETAIL
ROMANIA	HOSPITAL
ROMANIA	RETAIL
RUSSIA	HOSPITAL
RUSSIA	RETAIL
SAUDI ARABIA	HOSPITAL
SAUDI ARABIA	RETAIL
SERBIA	COMBINED
SINGAPORE	COMBINED
SLOVAK REPUBLIC	HOSPITAL
SLOVAK REPUBLIC	RETAIL
SLOVENIA	COMBINED
SOUTH AFRICA	COMBINED
SOUTH KOREA	HOSPITAL
SOUTH KOREA	RETAIL
SPAIN	HOSPITAL
SPAIN	RETAIL
SWEDEN	COMBINED

SWITZERLAND	HOSPITAL
SWITZERLAND	RETAIL
TAIWAN	HOSPITAL
TAIWAN	RETAIL
THAILAND	HOSPITAL
THAILAND	RETAIL
TUNISIA	HOSPITAL
TUNISIA	RETAIL
TURKEY	HOSPITAL
TURKEY	RETAIL
UK	HOSPITAL
UK	RETAIL
USA	HOSPITAL
USA	RETAIL
VIETNAM	HOSPITAL
VIETNAM	RETAIL

Table A.2 WHO essential medicines included in analysis

AMPHOTERICIN B
MICONAZOLE
NATAMYCIN
TETRACYCLINE
METRONIDAZOLE
CLOTRIMAZOLE
DOXYCYCLINE
DEXAMETHASONE
HYDROCORTISONE
EPINEPHRINE
ACETYLSALICYLIC ACID
RANITIDINE
MISOPROSTOL
OMEPRAZOLE
ATROPINE
METOCLOPRAMIDE
ONDANSETRON
LACTULOSE
MANNITOL
NALOXONE
NYSTATIN
VANCOMYCIN
LOPERAMIDE

PREDNISOLONE
BETAMETHASONE
BUDESONIDE
BECLOMETASONE
SULFASALAZINE
METFORMIN
GLICLAZIDE
ERGOCALCIFEROL
COLECALCIFEROL
NICOTINAMIDE
CALCIUM
WARFARIN
CLOPIDOGREL
TRANEXAMIC ACID
PHYTOMENADIONE
HYDROXOCOBALAMIN
FOLIC ACID
DEXTRAN
BLOOD PLASMA
GLUCOSE
DIGOXIN
LIDOCAINE
IBUPROFEN
METHYLDOPA
HYDRALAZINE
HYDROCHLOROTHIAZIDE
FUROSEMIDE
SPIRONOLACTONE
AMILORIDE
TETRACAINE
PROPRANOLOL
TIMOLOL
BISOPROLOL
AMLODIPINE
NIFEDIPINE
VERAPAMIL
ENALAPRIL
LOSARTAN
SIMVASTATIN
GRISEOFULVIN
FLUCONAZOLE

TERBINAFINE	
FLUCYTOSINE	
CHLORAMPHENICOL	
GENTAMICIN	
MUPIROCIN	
AMIKACIN	
ACICLOVIR	
POVIDONE-IODINE	
IODINE	
POTASSIUM PERMANGANATE	
ETHANOL	
RETINOL	
CLINDAMYCIN	
ERYTHROMYCIN	
DAPSONE	
EFLORNITHINE	
IVERMECTIN	
ACETIC ACID	
ERGOMETRINE	
LEVONORGESTREL	
MEDROXYPROGESTERONE	
ETONOGESTREL	
OXYTOCIN	
FLUDROCORTISONE	
PROPYLTHIOURACIL	
GLUCAGON	
AMPICILLIN	
AMOXICILLIN	
PENICILLIN V	
PENICILLIN G	
CLOXACILLIN	
CEFALEXIN	
CEFAZOLIN	
CEFOTAXIME	
CEFTRIAXONE	
CEFIXIME	
SULFADIAZINE	
SULFAMETHOXAZOLE + TRIMETHOPRIM	
CLARITHROMYCIN	
AZITHROMYCIN	
OFLOXACIN	

CIPROFLOXACIN
NITROFURANTOIN
SPECTINOMYCIN
ITRACONAZOLE
VORICONAZOLE
RIFAMPICIN
RIFABUTIN
ISONIAZID
PYRAZINAMIDE
ETHAMBUTOL
ISONIAZID + PYRIDOXINE + RIFAMPICIN
ETHAMBUTOL + ISONIAZID + PYRAZINAMIDE + RIFAMPICIN
CLOFAZIMINE
RIBAVIRIN
VALGANCICLOVIR
RITONAVIR
ATAZANAVIR
DARUNAVIR
SIMEPREVIR
ZIDOVUDINE
LAMIVUDINE
ABACAVIR
TENOFOVIR DISOPROXIL
ENTECAVIR
NEVIRAPINE
EFAVIRENZ
ABACAVIR + LAMIVUDINE
LAMIVUDINE + NEVIRAPINE + ZIDOVUDINE
EFAVIRENZ + EMTRICITABINE + TENOFOVIR DISOPROXIL
LOPINAVIR + RITONAVIR
EFAVIRENZ + LAMIVUDINE + TENOFOVIR DISOPROXIL
EMTRICITABINE + TENOFOVIR ALAFENAMIDE
RALTEGRAVIR
DOLUTEGRAVIR
DACLATASVIR
SOFOSBUVIR
DASABUVIR
LEDIPASVIR + SOFOSBUVIR
OMBITASVIR + PARITAPREVIR + RITONAVIR
IMMUNOGLOBULIN ANTI-CORYNEBACTERIUM DIPHTHERIAE TOXIN
IMMUNOGLOBULIN ANTIVENOM SNAKES

VACCINE, CHOLERA
VACCINE, PNEUMOCOCCAL
VACCINE, TETANUS
VACCINE, TYPHOID
VACCINE, JAPANESE ENCEPHALITIS
VACCINE, INFLUENZA
VACCINE, MEASLES
VACCINE, MUMPS
VACCINE, RABIES
VACCINE, ROTAVIRUS
VACCINE, RUBELLA
VACCINE, VARICELLA ZOSTER
VACCINE, YELLOW FEVER LIVE
FLUOROURACIL
VACCINE, TUBERCULOSIS
PENICILLAMINE
SUXAMETHONIUM
VECURONIUM BROMIDE
ATRACURIUM BESILATE
ALLOPURINOL
ISOFLURANE
FENTANYL
KETAMINE
PROPOFOL
BUPIVACAINE
MORPHINE
PARACETAMOL
PHENYTOIN
CARBAMAZEPINE
VALPROIC ACID
LAMOTRIGINE
BIPERIDEN
LEVODOPA
CHLORPROMAZINE
FLUPHENAZINE
HALOPERIDOL
RISPERIDONE
DIAZEPAM
MIDAZOLAM
CLOMIPRAMINE
AMITRIPTYLINE

FLUOXETINE
NEOSTIGMINE
PILOCARPINE
CHLOROQUINE
PRIMAQUINE
QUININE
MEFLOQUINE
ARTEMETHER
ARTESUNATE
ARTEMETHER + LUMEFANTRINE
BENZNIDAZOLE
SODIUM STIBOGLUCONATE
NIFURTIMOX
PRAZIQUANTEL
MEBENDAZOLE
ALBENDAZOLE
PYRANTEL
LEVAMISOLE
PERMETHRIN
BENZYL BENZOATE
XYLOMETAZOLINE
IPRATROPIUM BROMIDE
SALBUTAMOL
BUDESONIDE + FORMOTEROL
ACETYLCYSTEINE
CODEINE
CYCLIZINE
LORATADINE
ACETAZOLAMIDE
LATANOPROST
TROPICAMIDE
FLUORESCEIN
PROTAMINE
OXYGEN
TUBERCULIN TEST
IOHEXOL
CHARCOAL
ETHINYLESTRADIOL + LEVONORGESTREL
RIBOFLAVIN
ETHINYLESTRADIOL + NORGESTIMATE
ELECTROLYTE SOLUTIONS

SENNA
PYRIDOXINE
ULIPRISTAL ACETATE
THIAMINE
ESTRADIOL + NORETHISTERONE
ZINC
NITROGLYCERIN
ENOXAPARIN SODIUM
SENNA + SENNOSIDES A&B
GLIMEPIRIDE + PIOGLITAZONE
EMPAGLIFLOZIN + LINAGLIPTIN
ALOGLIPTIN + METFORMIN
CANAGLIFLOZIN + METFORMIN
DAPAGLIFLOZIN + METFORMIN
EMPAGLIFLOZIN + METFORMIN
GEMIGLIPTIN + METFORMIN
LINAGLIPTIN + METFORMIN
METFORMIN + SAXAGLIPTIN
METFORMIN + SITAGLIPTIN
METFORMIN + VILDAGLIPTIN
ALOGLIPTIN + PIOGLITAZONE
VACCINE, TICK BORNE ENCEPHALITIS
ETHAMBUTOL + ISONIAZID + PYRAZINAMIDE + RIFAMPICIN + STREPTOMYCIN
ETHAMBUTOL + ISONIAZID + RIFAMPICIN
ISONIAZID + PYRAZINAMIDE + RIFAMPICIN
ISONIAZID + PYRIDOXINE
ISONIAZID + RIFAMPICIN
ADENOSINE TRIPHOSPHATE + ARGININE + COENZYME A + DEXTRAN + INSULIN HUMAN BASE
ADENOSINE TRIPHOSPHATE + ARGININE + COENZYME A + DEXTRAN + INSULIN UNSPECIFIED BASE
ADENOSINE TRIPHOSPHATE + COENZYME A + INSULIN HUMAN BASE
ADENOSINE TRIPHOSPHATE + COENZYME A + INSULIN UNSPECIFIED BASE
INSULIN ASPART
INSULIN ASPART + INSULIN ASPART PROTAMINE CRYSTALLINE
INSULIN ASPART + INSULIN DEGLUDEC
INSULIN BOVINE BASE
INSULIN BOVINE ISOPHANE
INSULIN BOVINE PROTAMINE ZINC
INSULIN BOVINE ZINC SUSPENSION (COMPOUND)
INSULIN BOVINE ZINC SUSPENSION (UNSPECIFIED) + INSULIN PORCINE ZINC SUSPENSION (UNSPECIFIED)
INSULIN DEGLUDEC

INSULIN DEGLUDEC + LIRAGLUTIDE
INSULIN DETEMIR
INSULIN GLARGINE
INSULIN GLARGINE + LIXISENATIDE
INSULIN GLULISINE
INSULIN HUMAN BASE
INSULIN HUMAN BASE + INSULIN HUMAN ISOPHANE
INSULIN HUMAN ISOPHANE
INSULIN LISPRO
INSULIN LISPRO + INSULIN LISPRO PROTAMINE
INSULIN PORCINE BASE
INSULIN PORCINE BASE + INSULIN PORCINE ISOPHANE
INSULIN PORCINE BASE + INSULIN PORCINE PROTAMINE ISOPHANE
INSULIN PORCINE ISOPHANE
INSULIN UNSPECIFIED PROTAMINE ZINC
VACCINE, HUMAN PAPILLOMAVIRUS (HPV) TYPE-16 & 18
VACCINE, HUMAN PAPILLOMAVIRUS (HPV) TYPE-6,11,16 & 18
VACCINE, HUMAN PAPILLOMAVIRUS TYPE-6,11,16,18,31,33,45,52,58
VACCINE, MENINGOCOCCAL POLYSACCHARIDE AND OMV
VACCINE,MENINGOCOCCAL C CONJUGATE
VACCINE,MENINGOCOCCAL CONJUGATE

Table A.3 WHO essential medicines not available in IQVIA Analytics Link

POTASSIUM CHLORIDE
PAROMOMYCIN
ARTESUNATE AND AMODIAQUINE
SODIUM NITRITE
BARIUM SULFATE CONTAINING X-RAY CONTRAST MEDIA
CHLOROXYLENOL
BARIUM SULFATE WITHOUT SUSPENDING AGENTS
METHYLPHENOBARBITAL
HEPATITIS A, INACTIVATED, WHOLE VIRUS
ETHAMBUTOL AND ISONIAZID
RIFAMPICIN AND ISONIAZID
MELARSOPROL
METHYLTHIONINIUM CHLORIDE
CALCIUM GLUCONATE
DRUGS USED IN NICOTINE DEPENDENCE
TRICLABENDAZOLE
VAGINAL RING WITH PROGESTOGEN AND ESTROGEN

POLIOMYELITIS VACCINES
DILOXANIDE
HALOTHANE
DIPHTHERIA VACCINES
NITROUS OXIDE
PRUSSIAN BLUE
TUBERCULOSIS DIAGNOSTICS
CHLORINE CONTAINING PRODUCTS
SILVER SULFADIAZINE
BENZATHINE BENZYLPENICILLIN
TETANUS ANTITOXIN
LIDOCAINE, COMBINATIONS
PLASTIC IUD WITH COPPER
ZIDOVUDINE AND LAMIVUDINE
ARTESUNATE AND PYRONARIDINE
PENTAMIDINE ISETHIONATE
HEPATITIS VACCINES
MAGNESIUM SULFATE
DOCUSATE SODIUM
DIETHYLCARBAMAZINE
ORAL REHYDRATION SALT FORMULATIONS
SELENIUM SULFIDE
HEPATITIS B, PURIFIED ANTIGEN
SODIUM CHLORIDE
MILTEFOSINE
PLASTIC IUD WITH PROGESTOGEN
POTASSIUM IODIDE
SURAMIN SODIUM
BARIUM SULFATE WITH SUSPENDING AGENTS
ARTESUNATE AND MEFLOQUINE
NICLOSAMIDE
DICHLOROBENZYL ALCOHOL
AMODIAQUINE
PROGUANIL
RIFAPENTINE

Table A.4 covariates considered

Covariate	Transformation
DTP3 coverage proportion	Logit
Gross domestic product per capita	Natural log
Lag-distributed income per capita	Natural log
Healthcare access and quality index (HAQI)	Logit
Primary health care spending per capita	Natural log
Primary health care spending as a share of total health expenditure	Logit
Primary health care spending excluding drugs per capita	Natural log
Primary health care spending excluding drugs as a share of total health expenditure	Logit
Primary health care spending excluding prescription drugs per capita	Natural log
Primary health care spending excluding prescription drugs as a share of total health expenditure	Logit
Health spending in a hospital setting (HP1) per capita	Natural log
Health spending in a hospital setting (HP1) as a share of total health expenditure	Logit
Health spending from providers of medical goods (HP5) per capita	Natural log
Health spending from providers of medical goods (HP5) as a share of total health expenditure	Logit

Table A.5 Covariates selected for each model

Dependent variable	Covariates selected
Total prescription drug spending on essential medicines as a proportion of total prescription drug spending	Gross domestic product per capita, HAQI, primary health care spending excluding prescription drugs as a share of total health expenditure, random effects on GBD region
Prescription drug spending on essential medicines in hospitals as a fraction of total prescription drug spending in hospitals	Primary health care spending excluding prescription drugs as a share of total health expenditure, health spending from providers of medical goods (HP5) as a share of total health expenditure
Prescription drug spending on essential medicines in retail setting as a fraction of total prescription drug spending in retail setting	Primary health care spending excluding prescription drugs as a share of total health expenditure, HAQI, health spending from providers of medical goods (HP5) as a share of total health expenditure

B. Detailed methods for estimating inpatient vaginal delivery expenditures

SUMMARY

To incorporate inpatient vaginal delivery expenditures in our estimates of PHC, we derived country-specific volume and cost of inpatient vaginal and Cesarean-section delivery for all countries between 2000 and 2017. Although the cesarean section estimates were not included in our final definition of PHC, they were used as part of the process of estimating the cost and volume of inpatient vaginal delivery. The total number, or volume, of births by delivery method was drawn from 1,043 country-years of data from the Demographic and Health Surveys, Multiple Indicator Cluster Surveys, and online databases provided by the OECD, the Canadian Institute for Health Information, and the United States' Healthcare Cost and Utilization Project.[5–10] 160 country-years of cost data were drawn from published literature, and three databases: Healthcare Cost and Utilization Project , Canadian Institute for Health Information , and Health Care Cost Institute.[7,8,11]

With these data we estimated the proportion of all deliveries that were inpatient vaginal deliveries and cesarean sections using Spatio-Temporal Gaussian Process Regression (ST-GPR).[3] We multiplied the modeled proportions by the number of births from the Global Burden of Disease study to estimate the total number of inpatient vaginal and cesarean deliveries by country and year.[12] In order to estimate the cost of inpatient vaginal deliveries, we found only 33 cost estimates specifically for inpatient vaginal deliveries. However, the cost of cesarean sections was found for 160 country-years. We first used ST-GPR to model a complete time-series of cesarean section costs. Then based country-years where both vaginal and cesarean section costs were available, we estimated the ratio of the inpatient vaginal to cesarean cost of delivery [for every country-year in the study]. This modeled ratio was then multiplied by the predicted costs of cesarean section to estimate the cost of vaginal delivery for each country and year. Finally, we multiplied the predicted volume and cost estimates to produce estimates of inpatient vaginal delivery expenditures.

INTRODUCTION

In order to estimate spending on inpatient vaginal deliveries, we utilized a cost * volume approach. We estimated the volume and unit cost of inpatient vaginal deliveries for 195 countries from 2000-2017, and then multiplied the predicted volume and cost for each country-year. This framework is illustrated below in equation 1.

Equation 1.

$$Spending\ on\ \widehat{Inpatient}\ Vaginal\ Delivery$$
$$= N\ Inpatient\ \widehat{Vaginal}\ Deliveries * Cost\ of\ \widehat{Inpatient\ V}aginal\ Delivery$$

In the sections that follow, we outline (1) our methods for estimating the volume of inpatient vaginal deliveries, (2) the methods used to estimate the unit cost of inpatient vaginal deliveries, and (3) how we combined these predictions to produce our final estimates of spending on inpatient vaginal delivery.

ESTIMATING VOLUME OF INPATIENT DELIVERY

Methods

To estimate the number of inpatient vaginal deliveries for 195 countries from 2000 to 2017, we ran three models to produce complete time series of the following proportions: the proportion of all deliveries that are (1) vaginal deliveries in an inpatient setting (2) cesarean sections in an inpatient setting, and (3) either delivery type in an inpatient setting. *The second and third fractions were not directly used, but were modeled in order to give strength to the predictions of the first through a process of raking.* In order to estimate the total number of inpatient vaginal deliveries, the predicted fractions from the raked vaginal model were multiplied by the total number of births from the Global Burden of Disease study.[12] This calculation is presented below in equation 2.

Equation 2

$$\widehat{N\ Inpatient\ Deliveries} = \frac{\widehat{N\ Inpatient\ Deliveries}}{N\ Deliveries} * Total\ Births$$

<u>Sources of data</u>

We extracted data from the following sources:

- Demographic and Health Surveys (DHS)
- Multiple Indicator Cluster Surveys (MICS)
- Online databases provided by the Organisation for Economic Co-operation and Development (OECD)
- Canadian Institute for Health Information (CIHI)
- Healthcare Cost and Utilization Project (HCUP)

Below in table B.1, we present the number of country-years of data by component that we used for each source. In the following sections, we briefly outline the differing ways in which we incorporated data from each source.

Table B.1

	Cesarean section	Inpatient Vaginal delivery	Total Inpatient delivery
**DHS**	457	457	457
**MICS**	126	0	0
**OECD**	448	0	539
**CIHI**	5	5	5
**HCUP**	7	7	7

<u>DHS</u>

Using the GHDx, we identified 86 DHS surveys with relevant data. For each birth year reported within each survey, we calculated the weighted proportion of all deliveries that were (1) cesarean sections in a hospital setting with a length of

stay greater than 24 hours (2) vaginal deliveries in a hospital setting with a length of stay greater than 24 hours, and (3) any delivery type that took place hospital setting with a length of stay greater than 24 hours. When two surveys contained data for the same year, we dropped the data point with fewer observations. Finally, we removed all country-years of data with 140 or fewer observations. Visual inspections made clear that country years with sample sizes below this threshold were inconsistent with the rest of the data and appeared not to be representative of the broader population. This resulted in a removal of 12% of the data. However, because these data points only occurred on the ends of a survey's time range, this did not prevent us from using any of the identified surveys.

MICS

We utilized 55 MICS surveys with relevant data within the GHDx (http://ghdx.healthdata.org/). Although we identified MICS data on delivery location and whether the birth was a cesarean section, we were not able to identify data on the length of stay in the birthing facility. As such, we did not feel confident that this data had the capacity to accurately represent inpatient vaginal deliveries or total inpatient deliveries, especially since some encoded delivery locations were somewhat vague (e.g. "Hospital/clinic/health center"). However, since the surgical nature of cesarean sections implies that patients remain in a health facility for at least 24 hours, we used MICS surveys to calculate, for each birth year, the weighted proportion of all deliveries that were cesarean sections in a hospital setting. Finally, to establish consistency between the treatment of MICS and DHS, we removed all country years with 140 or fewer observations. This removed 34% of the data points. However, because these data points only occurred on one end of a survey's time range, this only prevented us from utilizing 3 of the identified surveys.

OECD

OECD databases provided 448 and 539 country-years of data on the rates of in-hospital cesarean sections and the total number of inpatient deliveries, respectively. Because these data were drawn from distinct OECD sources, we did not subtract these data to calculate the number of inpatient vaginal deliveries. Cesarean section rates were drawn directly from the OECD online database [6]. We used OECD data on hospital discharges to calculate the rate of inpatient deliveries[5]. Specifically, we summed the number of discharges that were assigned one of the following diagnostic related groups; "Complications of pregnancy predominantly during labour and delivery", "Single spontaneous delivery", and "Other delivery." In order to convert these sums to proportions, we divided them by the number of births from the Global Burden of Disease study. Finally, we manually outliered Norway's total inpatient delivery data from 2011-2017, as these data represent a definitional break from the more robust, pre-2011 time series.

CIHI and HCUP

CIHI and HCUP are hospital databases from Canada and the US. Data from CIHI was downloaded directly.[8] HCUP data was downloaded from the online querying system after the following selections were made: Get Quick Statistical Tables -> Inpatient -> National -> Do you want data on a specific diagnosis or procedure? : Yes -> MS-DRG -> Detailed Tables [7]. Within each database, we summed the number of cesarean sections and vaginal deliveries by year and divided by these numbers by the number of births from the Global Burden of Disease study. We then summed these proportions to calculate the proportion of all deliveries that took place in an inpatient setting.

Covariate Selection

Once we compiled data from the above sources, we split the data into the parts, corresponding to the three fractions outlined above: the proportion of all deliveries that are (1) vaginal deliveries in an inpatient setting (2) cesarean sections

in an inpatient setting, and (3) any delivery type in an inpatient setting. These fractions were modeled in logit space such that the predictions for each would remain bound between 0 and 1. Each of these fractions was modeled individually, and we performed covariate selection to identify the best set of covariates for each model.

In order to select each model's covariates, the following process was undertaken for each of the three fractional dependent variables outlined above: We first conducted a lasso regression to determine which covariates were least correlated, conditional on other covariates, with the dependent variable. Covariates with an estimated coefficient of zero were removed from the set of possible covariates. We then used linear mixed effects regression to assess all possible models based on combinations of the remaining covariates. Next, we selected the intersection of the 1000 models with the lowest Akaike information criterion (AIC) and the 1000 models with the lowest Bayesian information criterion (BIC). Finally, we completed a 10-fold cross-validation with out-of-sample predictions each of these remaining models. We selected the best model based on out-of-sample root mean squared error. Model selection considered the following covariates for inclusion in each model: Antenatal Care (4 visits) Coverage (proportion), In-Facility Delivery (proportion), Lag distributed income per capita, Skilled Birth Attendance (proportion), Total Fertility Rate, Healthcare access and quality index, Maternal Education (years per capita), and Socio-demographic Index. All covariates were drawn from the Global Burden of Disease study. Covariates bound between 0 and 1 were logit transformed, while the others were log transformed. The final selected covariates for each delivery fraction are shown below in Table B.2. Random effects were determined a priori.

Table B.2

	**Selected Covariates**
**Cesarean section**	Lag distributed income per capita, Maternal Education (years per capita), Antenatal Care (4 visits) Coverage (proportion), Skilled Birth Attendance (proportion), Socio-demographic Index, and In-Facility Delivery (proportion), with random effects at the country, region, and super region levels.
**Inpatient Vaginal delivery**	Lag distributed income per capita, Maternal Education (years per capita), and Socio-demographic Index, with random effects at the region, and super region levels.
**Total Inpatient delivery**	Skilled Birth Attendance (proportion), Healthcare access and quality index, and In-Facility Delivery (proportion), with random effects at the country, region, and super region levels.

Modeling

We modeled each of these logit-transformed delivery proportions (vaginal inpatient, cesarean inpatient, and total inpatient) using spatiotemporal Gaussian process regression (ST-GPR). ST-GPR generates a complete time-series of data for a set of locations, follows the data where it is available and borrows strength across time, geographic regions, and covariates' predictive power when data is not available. STGPR has three primary steps. First, a linear mixed effects model is run with a given set of predictors. These first-stage models are displayed above in Table B.2. Predictions from the first step provide the general trend within the data. In the second step, spatiotemporal patterns are estimated by applying a series of spatiotemporal weights to average the residuals of the first step linear model. These spatiotemporal patterns are then added to the linear predictions to generate spatiotemporally smoothed predictions. Finally, the smoothed predictions are passed as the mean function to a Gaussian process regression along with the data to produce

final ST-GPR predictions. For every country-year for each model, 1,000 draws were generated from the Gaussian process regression model and used in subsequent calculations.

Raking

In order to strengthen the predictions of the volume of inpatient vaginal delivery, we raked the modeled vaginal and cesarean fractions such that they summed to the modeled inpatient delivery fraction. Raking was done at the draw level, such that the sum of draw 1 from the vaginal and cesarean proportions was forced equal draw 1 of the total inpatient delivery envelope. Draws were raked proportionally such that the original relationship between the vaginal and cesarean fractions was preserved.

Final Calculation

Once the country and year specific inpatient delivery fractions had been raked, we multiplied them by the total number of births from the Global Burden of Disease study to obtain the total number of inpatient vaginal deliveries, as illustrated in Equation 2. This multiplication was done at the draw level. Because these estimates were raked, the modeled total number of inpatient deliveries is equal to the predicted sum of the number of inpatient vaginal and cesarean deliveries.

ESTIMATING THE COST OF INPATIENT VAGINAL DELIVERY

Summary of methods

In order to estimate the cost of inpatient vaginal deliveries for 195 countries from 2000 to 2017, we conducted a literature review and extracted cost data from the existing research. Because data on the cost of inpatient vaginal deliveries was sparse, we leveraged more data on the cost of cesarean sections. Specifically, we modeled the extracted cesarean section costs to produce a complete time series, and then, using extracted data on the cost of vaginal deliveries in inpatient settings, scaled the modeled cesarean costs to estimate the cost of inpatient vaginal delivery.

Sources of data

Overall, we utilized 160 and 33 country-years of cesarean section and inpatient vaginal delivery unit costs, respectively. This data was drawn from 10 research articles and 3 databases.[7,8,11,13–22] When aggregate volume and cost data were provided, we divided the two to estimate unit costs. When disaggregated unit costs were provided along with data weights (e.g. the cost of cesarean sections with and without complications, and the number or proportion of each), we took the weighted average of the disaggregate costs.

Currency Conversion

All final estimates are presented in 2017 United States dollars (USD). As such, it was necessary to convert all extracted cost data to 2017 USD. Data sources reported spending in either nominal local currency units (LCU) or nominal USD. To convert nominal LCU to USD, we applied deflators to nominal LCU to inflate to 2017 LCU. We then applied exchange rates to produce 2017 USD. When LCU were not reported, we extracted reported expenditure in nominal USD, applied corresponding nominal exchange rates to produce nominal LCU, inflated nominal LCU to 2017 LCU with deflators, and finally exchanged 2017 LCU to 2017 USD.

Scaling data from Gibbons et al.

Gibbons et al. (2010) [20] was our main source of cesarean section costs, especially for low- and middle-income countries. However, this research only provided the additional cost of caesarean section over and above the cost of vaginal delivery, and as such, "none of the routine costs associated with antenatal care visits were included, nor were other services that would be considered part of normal vaginal delivery" (p.6). In order to use this data in our models, it was necessary to scale the raw data. We assumed that the data from Gibbons et al. was equal to the cost of cesarean section (CS) minus the cost of vaginal delivery (VD).

In order to scale this data to the full cost of cesarean sections, we multiplied this raw data by the scalar $\rho = \frac{CS\ cost}{CS\ cost - VD\ cost}$, or the ratio between the full cost of cesarean sections and the additional cost of cesarean sections. We first calculated this ratio using data from sources which reported the full unit cost of both cesarean section and inpatient vaginal delivery. This calculation yielded 33 data points. We then ran a mixed effects linear regression on these 33 data points using lag-distributed income (LDI) from the Global Burden of Disease study as a covariate with random effects at the country level. Next, we used the resulting model along with data on LDI to predict this ratio, $\hat{\rho}$, for all country-years of data for which we had extracted data from Gibbons et al. Finally, we multiplied the raw data from Gibbons et al. by these predicted ratios to scale the additional cost of cesarean sections up to the full cost. This calculation is illustrated below in Equation 3, where $\hat{\rho}$ is the predicted ratio described above.

Equation 3

$$CS\ \widehat{cost}_{Gibbons} = Gibbons\ raw\ data * \hat{\rho}$$

After scaling, we used visual inspection to outlier the costs from Iceland and Estonia, which were unreasonably high. The remaining 121 scaled data points from Gibbons et al. were appended to the rest of the extracted data, yielding a total of 160 data points on the cost of cesarean sections, spanning 151 country years

Caesarean Cost Modeling

The methodology for modeling cesarean section costs was similar to that used in our volume models. The dependent variable in the cesarean section cost model was the natural log of the unit cost of cesarean sections. Covariates for this model were identified using the same methods as previously described for the volume models. Model selection considered the following covariates for inclusion in each model: Antenatal Care (4 visits) Coverage (proportion), In-Facility Delivery (proportion), Lag distributed income per capita, Skilled Birth Attendance (proportion), Total Fertility Rate, Healthcare access and quality index, Inpatient Unit Costs, Maternal Education (years per capita), and Socio-demographic Index. Inpatient unit costs were drawn from Moses et al. (2018) [23], and all other covariates were drawn from the Global Burden of Disease study.[4] Covariates bound between 0 and 1 were logit transformed, while the others were log transformed. The final selected model used the following covariates: Antenatal Care (4 visits) Coverage (proportion), Socio-demographic Index, Maternal Education (years per capita), Inpatient Unit Costs, with random effects at the country, region, and super region level. To detect and remove the influence of outlier data points, we used the selected model to measure Cooke's distance for each data point. We excluded 7 data points whose Cook's distance was greater than 4/n, where n is the total number of data points in the model. After removing these outlier points, we ran ST-GPR on the data, using the selected model as the first stage of ST-GPR. The workings of ST-GPR are described above in the section on volume modeling. For every country-year, 1,000 draws were generated and used in subsequent calculations.

Calculating the Cost of Inpatient Vaginal Delivery

We did not identify sufficient data on the cost of inpatient vaginal deliveries to directly model these data. As such, similar to the way in which we scaled the data from Gibbons et al. (2010)[20], we used the available raw cost data to scale down the modeled cost of cesarean sections to the cost of inpatient vaginal delivery. Specifically, we calculated the ratio $\theta = \frac{VD\ cost}{CS\ cost}$ using data from sources which provided both cesarean and inpatient vaginal costs. This calculation yielded 33 data points. We then ran a mixed effects linear regression to predict this ratio with lag-distributed income (LDI) from the Global Burden of Disease study as a covariate and random effects at the country level. Next, we used this model and data on LDI to predict the ratio $\hat{\theta}$ for all 195 countries from 2000 to 2017. Finally, for each country-year, we applied this ratio to scale down the draws from the caesarean section cost model to the cost of inpatient vaginal delivery. This calculation is illustrated below in Equation 4.

Equation 4

$$\widehat{VD\ cost} = \widehat{CS\ cost} * \hat{\theta}$$

TOTAL SPENDING ON INPATIENT VAGINAL DELIVERY

Calculating Total Spending

Before final calculations were done, the volume and cost models were vetted to ensure reasonable model fit using a custom Shiny app built in R. Once the best model versions were identified, the following steps were taken: The raked predictions of the number of inpatient vaginal deliveries were multiplied by the predicted costs of inpatient vaginal deliveries to yield total spending on inpatient vaginal deliveries, for each country-year. These calculations were done at the draw level to propagate uncertainty.

Adjusting the Estimates to Correspond with NHA Estimates

In order to ensure that our estimates correspond with existing estimates of inpatient spending, we compared the estimates of total inpatient delivery spending (cesarean + inpatient vaginal) to the predicted total inpatient spending from Schneider et al.[24] (In order to generate estimates of total inpatient spending, we multiplied the modeled cesarean volume and cost estimates produced above and summed these with the modeled inpatient vaginal delivery estimates). In order to compare to the NHA envelope, we divided the predicted spending on inpatient delivery by the predicted total inpatient spending. We identified, by visual inspection, that this proportion fell below 0.6 for almost all country years; however, in some countries, the proportion exceeded 1. As such, we applied the following correction to the estimates of delivery spending: For all country-years where the mean spending on inpatient delivery was higher than 60% of the total spending on inpatient care, we scaled down the draws of inpatient delivery spending such that (1) their new mean is 60% of all inpatient spending and (2) no delivery spending draws exceed the predicted total spending on inpatient care. This adjustment was only necessary for 5 countries: Bangladesh, Myanmar, Nepal, South Sudan, and Yemen. Finally, we proportionally raked the draws of spending on inpatient vaginal delivery and cesarean section such that they sum to this newly adjusted total. This yielded the final estimates of inpatient vaginal delivery spending which were used as a component of the spending on primary health care.

C. Comparison of estimates of primary health care expenditures versus country reported primary health care expenditures from national health accounts

Based upon the national health accounts (NHAs) reviewed by Schneider et al, we extracted all instances of reported primary health are (PHC) expenditures from NHA reports. As definitions of PHC expenditure by countries who create NHAs varies, we attempted to categorize these expenditures based upon if the definition focused on the types of facilities or the services provided. All extracted PHC expenditures were converted to per capita 2017 US dollars as to be comparable with the PHC estimates provided within the main article of this research. Figure C.1 presents the comparison of the extracted expenditures (x-axis) and the estimated PHC expenditures (y-axis, which consider all PHC services irrelevant of the provider). Figure C.2 presents the comparison of the extracted expenditures (x-axis) and the estimated PHC expenditures within ambulatory settings (y-axis). We can see from these two figures that the PHC expenditures reported by countries align more closely with the definition of PHC expenditures in an ambulatory setting. It is unclear if this alignment is due to countries consciously defining their PHC expenditure estimate to the ambulatory setting or more driven by lack of clarity of health PHC services provided outside of ambulatory settings.

Figure C.1

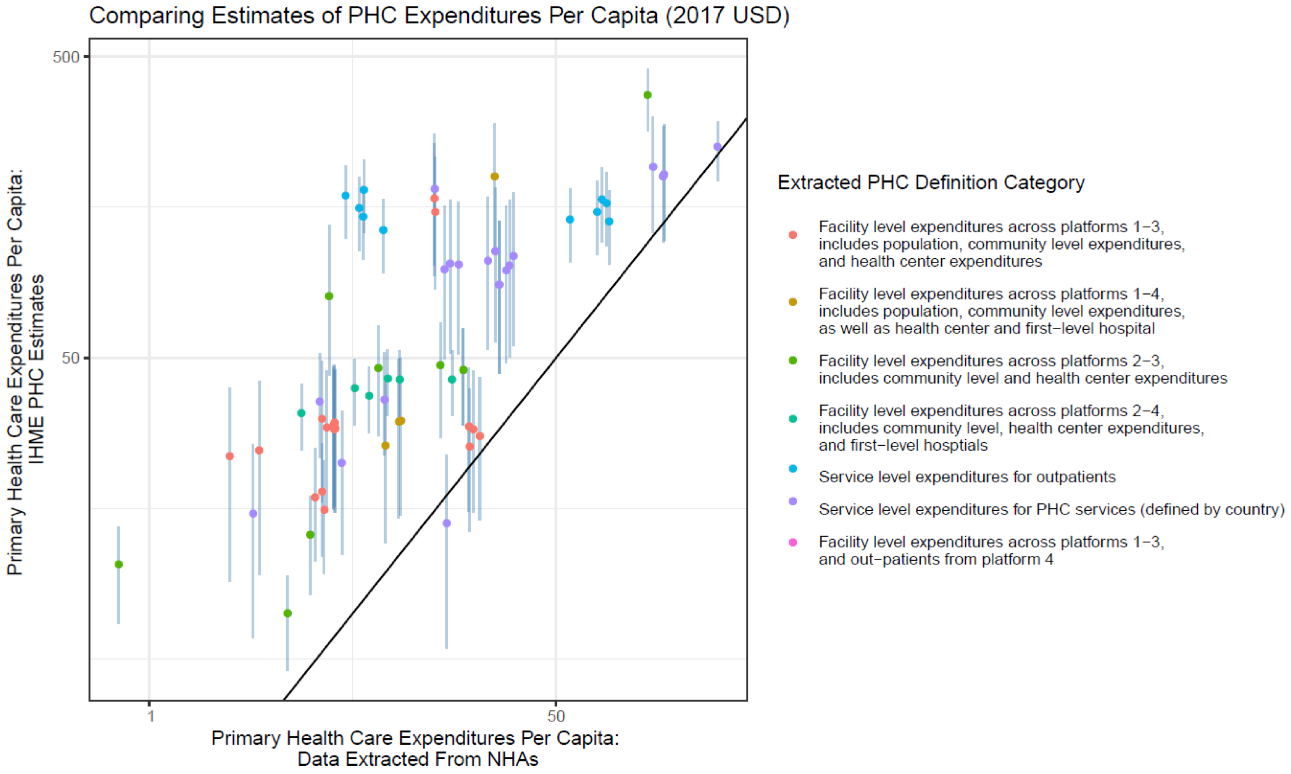

Figure C.2

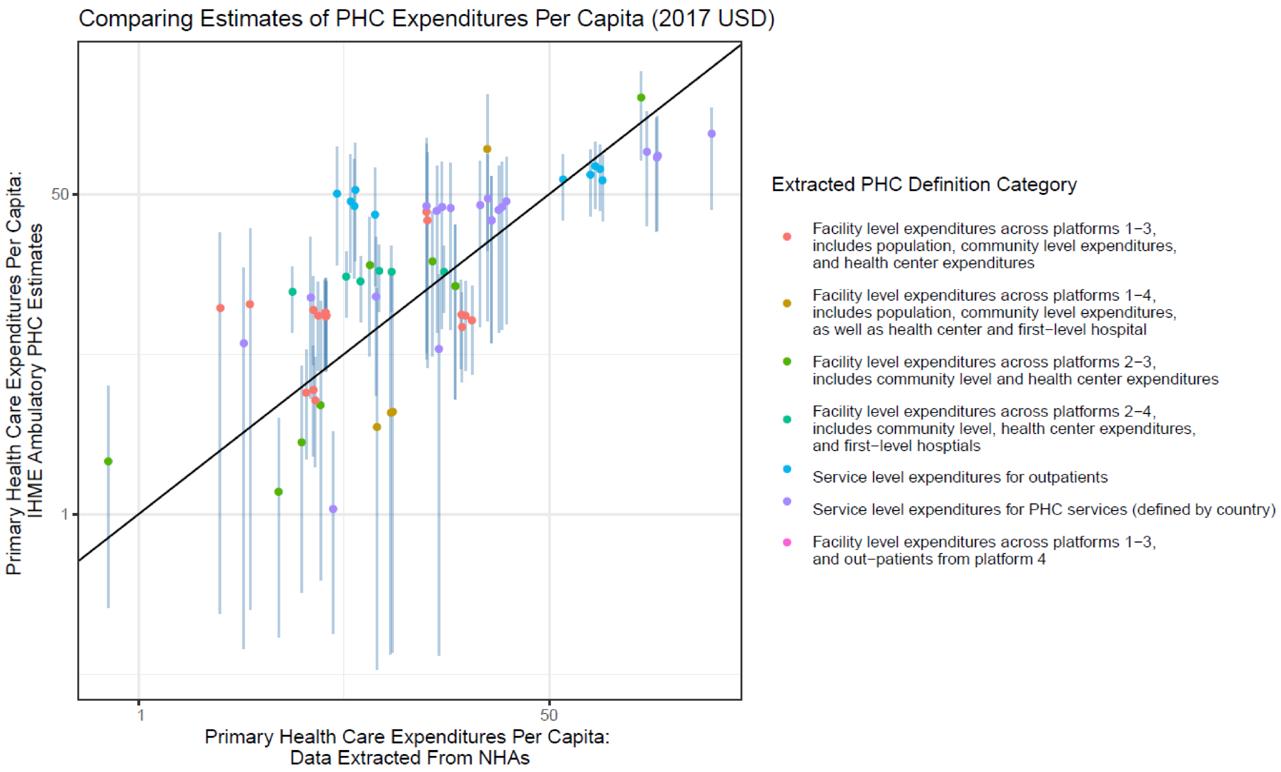
Comparing Estimates of PHC Expenditures Per Capita (2017 USD)
Primary Health Care Expenditures Per Capita:
IHME Ambulatory PHC Estimates
50
1
1
50
Primary Health Care Expenditures Per Capita:
Data Extracted From NHAs
Extracted PHC Definition Category
Facility level expenditures across platforms 1−3, includes population, community level expenditures, and health center expenditures
Facility level expenditures across platforms 1−4, includes population, community level expenditures, as well as health center and first−level hospital
Facility level expenditures across platforms 2−3, includes community level and health center expenditures
Facility level expenditures across platforms 2−4, includes community level, health center expenditures, and first−level hosptials
Service level expenditures for outpatients
Service level expenditures for PHC services (defined by country)
Facility level expenditures across platforms 1−3, and out−patients from platform 4

D. Complete outcome/output fixed effects regression outputs

Low and Middle Income Countries: Fixed Effects: Health Outcomes

	Dependent variable:						
	All-age mortality	Communicable DALYs	Non-communicable DALYs	Diabetes Prevalence	Maternal mortality	Neonatal mortality	Under-5 mortality
PHC/THE	1.076	0.995	0.071	-0.846	-10.309***	-3.285	-0.120
	(0.819)	(1.329)	(0.391)	(0.388)	(1.681)	(1.124)	(1.167)
THE per capita (log)	-0.020	-0.065**	0.023**	-0.008	-0.028	-0.077***	-0.075**
	(0.012)	(0.019)	(0.006)	(0.006)	(0.026)	(0.017)	(0.020)
LDI per capita (log)	-0.150***	-0.297***	-0.121***	-0.010	-0.442***	-0.213***	-0.305***
	(0.019)	(0.039)	(0.009)	(0.007)	(0.041)	(0.028)	(0.027)
Fertility rate (log)	-0.031	-0.063	-0.027	-0.030	-0.400***	-0.337***	-0.152*
	(0.026)	(0.044)	(0.014)	(0.013)	(0.064)	(0.041)	(0.050)
Hospital beds per 1000 (log)	-0.127***	0.082	-0.131***		-0.695***	-0.120	-0.057
	(0.027)	(0.052)	(0.017)		(0.085)	(0.054)	(0.046)
Education yrs. per capita (log)	-0.200***	-0.553***		0.164***	-0.226	-0.548***	-0.751***
	(0.037)	(0.064)		(0.020)	(0.093)	(0.074)	(0.081)
HIV Prevalence (log)	0.053***	0.069***	0.004	0.027***	-0.003	-0.106***	-0.057***
	(0.006)	(0.013)	(0.003)	(0.004)	(0.020)	(0.010)	(0.011)
Urbanicity (logit)	-0.084**	-0.158***	-0.031	-0.024*	-0.139	-0.075	-0.174***
	(0.023)	(0.037)	(0.011)	(0.007)	(0.052)	(0.027)	(0.040)
OOP as a share of THE (logit)		-0.006		-0.009	-0.036	0.003	0.004
		(0.012)		(0.003)	(0.016)	(0.009)	(0.010)
GHES as a share of THE (logit)	-0.0001	-0.025	0.006	0.008*	0.004	-0.044***	-0.027*
	(0.006)	(0.010)	(0.003)	(0.002)	(0.012)	(0.008)	(0.009)
Population over 65 (logit)	0.112***	0.145***	0.027	-0.050***	0.213***	-0.118**	0.055
	(0.023)	(0.035)	(0.010)	(0.012)	(0.048)	(0.033)	(0.045)
Observations	2,430	2,430	2,430	2,430	2,430	2,414	2,430
R^2	0.152	0.224	0.141	0.110	0.218	0.289	0.205
Adjusted R^2	0.092	0.169	0.080	0.046	0.162	0.238	0.148
Note:							*p<0.05; **p<0.01; ***p<0.001

Low and Middle Income Countries: Fixed Effects: Health Outputs

	Dependent variable:								
	HAQ Index	Health worker Density	Smoking Prevalence	UHC index	ANC4 coverage	ART Coverage	DTP3 coverage	Measles Vaccine Coverage	SBA coverage
PHC/THE	1.294*	-1.072	-0.954	-0.249	12.556***	-10.301	9.311	20.975***	-2.713
	(0.415)	(0.622)	(0.627)	(0.573)	(2.223)	(10.507)	(4.982)	(4.927)	(3.364)
THE per capita (log)	-0.010	0.129***	0.009	0.002	0.232***	0.552*	0.212*	0.560***	0.154*
	(0.007)	(0.009)	(0.011)	(0.010)	(0.036)	(0.176)	(0.069)	(0.080)	(0.051)
LDI per capita (log)	0.216***	0.289***	0.018	0.165***	0.474***	1.122**		-0.426*	0.941***
	(0.010)	(0.018)	(0.017)	(0.014)	(0.053)	(0.291)		(0.125)	(0.082)
Fertility rate (log)	-0.082***	-0.331***	0.136***	0.074**	-0.355***	1.816***	-0.362	-0.902**	-0.787***
	(0.016)	(0.025)	(0.027)	(0.020)	(0.082)	(0.408)	(0.242)	(0.256)	(0.098)
Hospital beds per 1000 (log)	0.077***	0.209***	0.088	-0.026	-0.123	0.687	-0.718*	-1.375***	1.037***
	(0.018)	(0.029)	(0.034)	(0.021)	(0.106)	(0.509)	(0.213)	(0.259)	(0.150)
Education yrs. per capita (log)	0.469***	-0.070	-0.010	0.202***	1.085***	5.917***	2.183***	2.273***	1.356***
	(0.028)	(0.044)	(0.045)	(0.033)	(0.123)	(0.674)	(0.273)	(0.349)	(0.143)
HIV Prevalence (log)	-0.008	0.025***	0.024**	-0.061***	0.147***	0.125	-0.004		0.079
	(0.005)	(0.006)	(0.007)	(0.005)	(0.026)	(0.115)	(0.048)		(0.027)
Urbanicity (logit)	0.103***		0.097***	0.065**	0.420***	-0.562	0.683***	0.380	-0.050
	(0.015)		(0.017)	(0.018)	(0.069)	(0.380)	(0.110)	(0.144)	(0.095)
OOP as a share of THE (logit)	-0.018**	-0.014	0.011	-0.008		0.355	-0.112	0.068	-0.092*
	(0.005)	(0.006)	(0.007)	(0.006)		(0.126)	(0.039)	(0.044)	(0.030)
GHES as a share of THE (logit)	-0.005	-0.002		-0.009	0.042	0.258		0.174***	0.144***
	(0.004)	(0.006)		(0.006)	(0.016)	(0.108)		(0.038)	(0.025)
Population over 65 (logit)	-0.060***	0.004	-0.081*	-0.162***	0.547***	-2.897***	0.135	-0.114	0.056
	(0.011)	(0.021)	(0.027)	(0.017)	(0.064)	(0.384)	(0.179)	(0.164)	(0.081)
Observations	2,430	2,430	2,430	2,430	2,430	2,430	2,430	2,430	2,430
R^2	0.503	0.429	0.049	0.281	0.252	0.138	0.072	0.082	0.263
Adjusted R^2	0.467	0.388	-0.019	0.230	0.199	0.076	0.007	0.017	0.210

Note: *p<0.05; **p<0.01; ***p<0.001

Low Income Countries: Fixed Effects: Health Outcomes

	Dependent variable:						
	All-age mortality	Communicable DALYs	Non-communicable DALYs	Diabetes Prevalence	Maternal mortality	Neonatal mortality	Under-5 mortality
PHC/THE	4.707	5.110	3.663***	0.120	-4.977	-6.343*	0.135
	(1.602)	(2.054)	(0.459)	(0.563)	(2.204)	(1.915)	(3.045)
THE per capita (log)	-0.010	-0.057	0.025	-0.017	0.0003	-0.162***	-0.219***
	(0.020)	(0.028)	(0.010)	(0.007)	(0.032)	(0.022)	(0.039)
LDI per capita (log)	-0.198***	-0.464***	-0.132***		-0.279**	-0.044	-0.219***
	(0.034)	(0.056)	(0.016)		(0.071)	(0.037)	(0.054)
Fertility rate (log)	0.174	0.379*		0.101*	0.051	0.259	0.222
	(0.090)	(0.112)		(0.033)	(0.148)	(0.133)	(0.186)
Hospital beds per 1000 (log)	-0.022	-0.253*	-0.127***	0.016	-0.283		-0.479***
	(0.051)	(0.074)	(0.030)	(0.020)	(0.098)		(0.089)
Education yrs. per capita (log)	0.355*	0.291	0.054		0.444*	-0.483***	0.373
	(0.111)	(0.126)	(0.036)		(0.130)	(0.081)	(0.234)
HIV Prevalence (log)	0.115***	0.153***	0.043***	0.050***	0.012	0.011	0.034
	(0.017)	(0.022)	(0.007)	(0.008)	(0.027)	(0.013)	(0.030)
Urbanicity (logit)	-0.854***	-0.856***			-0.703***	-0.544***	-0.876***
	(0.086)	(0.123)			(0.145)	(0.102)	(0.164)
OOP as a share of THE (logit)	-0.040**	-0.062*	-0.00000		-0.051	-0.034	-0.101***
	(0.011)	(0.018)	(0.005)		(0.017)	(0.013)	(0.022)
GHES as a share of THE (logit)	0.031*	0.011	0.011	0.004	0.016	0.005	0.042
	(0.010)	(0.017)	(0.004)	(0.004)	(0.017)	(0.012)	(0.014)
Population over 65 (logit)	0.158		-0.059	-0.047	-0.001	0.209*	0.128
	(0.074)		(0.025)	(0.025)	(0.112)	(0.066)	(0.182)
Observations	558	558	558	558	558	542	558
R^2	0.422	0.528	0.271	0.232	0.207	0.376	0.266
Adjusted R^2	0.355	0.474	0.189	0.149	0.115	0.303	0.181

Note: *p<0.05; **p<0.01; ***p<0.001

Low Income Countries: Fixed Effects: Health Outputs

	Dependent variable:								
	HAQ Index	Health worker Density	Smoking Prevalence	UHC index	ANC4 coverage	ART Coverage	DTP3 coverage	Measles Vaccine Coverage	SBA coverage
PHC/THE	-0.356	1.490	-0.613	-4.600**	25.842***	37.829	-20.041	12.309	-2.972
	(1.026)	(1.086)	(1.439)	(1.304)	(4.371)	(15.600)	(11.131)	(6.875)	(4.286)
THE per capita (log)	0.028	0.190***	-0.025	0.036	0.473***	1.080***	0.120	0.737***	0.160
	(0.015)	(0.014)	(0.023)	(0.020)	(0.059)	(0.175)	(0.145)	(0.106)	(0.077)
LDI per capita (log)	0.287***	0.187***	0.011	0.334***	0.752***	1.158*			1.423***
	(0.028)	(0.030)	(0.048)	(0.036)	(0.114)	(0.364)			(0.148)
Fertility rate (log)	0.312***		0.029	0.146	1.260***	2.134*	1.126	0.244	1.604***
	(0.056)		(0.074)	(0.054)	(0.280)	(0.645)	(0.459)	(0.322)	(0.296)
Hospital beds per 1000 (log)		-0.510***	-0.044	-0.127	0.341	1.874	0.320	2.193***	
		(0.056)	(0.074)	(0.083)	(0.294)	(0.783)	(0.471)	(0.399)	
Education yrs. per capita (log)	0.207	-0.128	0.276*	0.319**	1.743***				-1.013*
	(0.080)	(0.075)	(0.091)	(0.086)	(0.417)				(0.296)
HIV Prevalence (log)	-0.046**	-0.032		-0.140***	-0.083				-0.082
	(0.013)	(0.012)		(0.020)	(0.070)				(0.053)
Urbanicity (logit)	0.209	0.184**	-0.231		-1.394***	1.790	1.942**		2.685***
	(0.074)	(0.053)	(0.100)		(0.263)	(0.807)	(0.558)		(0.350)
OOP as a share of THE (logit)	0.016		-0.037	0.044**		0.436**	-0.102	0.422***	0.003
	(0.010)		(0.016)	(0.012)		(0.114)	(0.067)	(0.075)	(0.041)
GHES as a share of THE (logit)	-0.019	-0.010		0.028	0.112*	-0.239			
	(0.009)	(0.007)		(0.013)	(0.033)	(0.096)			
Population over 65 (logit)	0.072	-0.047		-0.095	1.359***	0.961	0.116	1.380*	0.547
	(0.034)	(0.034)		(0.051)	(0.174)	(0.560)	(0.384)	(0.402)	(0.186)
Observations	558	558	558	558	558	558	558	558	558
R^2	0.429	0.662	0.038	0.437	0.369	0.150	0.089	0.114	0.453
Adjusted R^2	0.363	0.625	-0.068	0.373	0.297	0.055	-0.009	0.021	0.392

Note: *p<0.05; **p<0.01; ***p<0.001

Lower Middle Income Countries: Fixed Effects: Health Outcomes

	Dependent variable:						
	All-age mortality	Communicable DALYs	Non-communicable DALYs	Diabetes Prevalence	Maternal mortality	Neonatal mortality	Under-5 mortality
PHC/THE	-2.925	-6.658**	-1.392	-1.227**	-4.550*	-4.320***	-3.892*
	(1.048)	(1.847)	(0.495)	(0.349)	(1.470)	(0.975)	(1.282)
THE per capita (log)	-0.023	-0.043	0.003	0.001	0.102	-0.039	-0.027
	(0.021)	(0.032)	(0.009)	(0.006)	(0.036)	(0.017)	(0.020)
LDI per capita (log)	-0.041	-0.170	-0.083***		-0.430***	-0.129***	-0.215***
	(0.038)	(0.062)	(0.014)		(0.048)	(0.028)	(0.028)
Fertility rate (log)	-0.061	-0.013		-0.096***	-0.324**	-0.256***	-0.110
	(0.048)	(0.096)		(0.020)	(0.086)	(0.058)	(0.060)
Hospital beds per 1000 (log)	-0.103		-0.015	-0.062	-0.181		0.142
	(0.040)		(0.022)	(0.032)	(0.079)		(0.061)
Education yrs. per capita (log)	-0.170		-0.040	0.151***	0.256	-0.635***	-0.875***
	(0.071)		(0.031)	(0.030)	(0.120)	(0.091)	(0.083)
HIV Prevalence (log)	0.058***	0.081***	-0.002	0.001	-0.018	-0.059***	-0.005
	(0.009)	(0.019)	(0.004)	(0.005)	(0.016)	(0.012)	(0.012)
Urbanicity (logit)	-0.062	-0.164	-0.001	-0.038	0.017	-0.087	-0.202**
	(0.045)	(0.092)	(0.024)	(0.019)	(0.060)	(0.055)	(0.057)
OOP as a share of THE (logit)		0.057	0.007	0.0004		-0.008	0.022
		(0.021)	(0.005)	(0.005)		(0.010)	(0.012)
GHES as a share of THE (logit)	0.007	0.022	0.007	0.003	0.037	-0.003	
	(0.008)	(0.017)	(0.003)	(0.003)	(0.013)	(0.009)	
Population over 65 (logit)	0.058	0.126	0.037	-0.085***	-0.244*	-0.195***	-0.054
	(0.039)	(0.070)	(0.022)	(0.020)	(0.078)	(0.040)	(0.053)
Observations	846	846	846	846	846	846	846
R^2	0.103	0.146	0.153	0.162	0.273	0.264	0.389
Adjusted R^2	0.018	0.066	0.073	0.083	0.204	0.195	0.332

Note: *p<0.05; **p<0.01; ***p<0.001

Lower Middle Income Countries: Fixed Effects: Health Outputs

	Dependent variable:								
	HAQ Index	Health worker Density	Smoking Prevalence	UHC index	ANC4 coverage	ART Coverage	DTP3 coverage	Measles Vaccine Coverage	SBA coverage
PHC/THE	0.486	-1.454	-1.737	1.708	13.403***	4.895	-17.829	15.894	10.090*
	(0.746)	(1.073)	(1.163)	(1.217)	(3.071)	(14.763)	(7.457)	(8.811)	(3.180)
THE per capita (log)	-0.061***	0.079***	-0.025	-0.058*	0.126	-0.057	-0.043	0.437	0.325***
	(0.012)	(0.019)	(0.020)	(0.019)	(0.077)	(0.350)	(0.151)	(0.148)	(0.077)
LDI per capita (log)	0.254***	0.317***	0.033	0.201***	0.501***	1.553	0.130	-0.314	
	(0.019)	(0.033)	(0.026)	(0.028)	(0.111)	(0.545)	(0.193)	(0.224)	
Fertility rate (log)	0.017	-0.582***	0.476***	0.210***		2.976***	-2.139**	-3.825***	-0.935***
	(0.028)	(0.047)	(0.059)	(0.044)		(0.636)	(0.548)	(0.564)	(0.187)
Hospital beds per 1000 (log)		0.152		0.038	-0.504	2.358			0.027
		(0.065)		(0.038)	(0.185)	(1.009)			(0.218)
Education yrs. per capita (log)	0.451***	-0.497***	0.063	0.019	0.598		1.569	-0.038	0.668
	(0.048)	(0.086)	(0.089)	(0.061)	(0.236)		(0.568)	(0.575)	(0.287)
HIV Prevalence (log)	0.024***		0.041**	-0.046***		-0.679**	0.045	0.108	-0.004
	(0.005)		(0.010)	(0.009)		(0.196)	(0.086)	(0.087)	(0.053)
Urbanicity (logit)	0.086	-0.156	0.109	0.159*	0.395			1.161**	0.493
	(0.036)	(0.053)	(0.062)	(0.050)	(0.153)			(0.324)	(0.175)
OOP as a share of THE (logit)	-0.050***	-0.025	0.004	-0.057***					0.103
	(0.007)	(0.015)	(0.013)	(0.010)					(0.039)
GHES as a share of THE (logit)	-0.003	-0.030		-0.034***	-0.021	0.489	0.185	0.197	0.021
	(0.005)	(0.010)		(0.008)	(0.027)	(0.184)	(0.080)	(0.067)	(0.028)
Population over 65 (logit)	0.016	0.066	0.028	-0.119*	0.787***	-2.383	-0.151	-0.431	0.276
	(0.024)	(0.047)	(0.048)	(0.038)	(0.144)	(0.846)	(0.308)	(0.294)	(0.191)
Observations	846	846	846	846	846	846	846	846	846
R^2	0.545	0.425	0.174	0.261	0.212	0.103	0.130	0.184	0.159
Adjusted R^2	0.502	0.370	0.097	0.190	0.140	0.020	0.051	0.108	0.079

Note: *p<0.05; **p<0.01; ***p<0.001

Upper Middle Income Countries: Fixed Effects: Health Outcomes

	Dependent variable:						
	All-age mortality	Communicable DALYs	Non-communicable DALYs	Diabetes Prevalence	Maternal mortality	Neonatal mortality	Under-5 mortality
PHC/THE	0.847	-1.078	0.476	-1.453	-24.442***	-3.664	-2.106
	(1.297)	(2.074)	(0.597)	(0.777)	(2.449)	(2.014)	(1.863)
THE per capita (log)	-0.006	-0.054	0.041***	-0.004	-0.130	-0.006	0.004
	(0.019)	(0.030)	(0.009)	(0.009)	(0.050)	(0.033)	(0.031)
LDI per capita (log)	-0.178***	-0.281**	-0.148***		-0.377***	-0.259***	-0.330***
	(0.029)	(0.074)	(0.018)		(0.088)	(0.059)	(0.048)
Fertility rate (log)	-0.086	-0.352***	-0.083**		-0.784***	-0.451***	-0.432***
	(0.033)	(0.067)	(0.022)		(0.109)	(0.075)	(0.069)
Hospital beds per 1000 (log)	-0.199***	0.088	-0.171***		-1.179***	-0.339***	-0.197*
	(0.037)	(0.070)	(0.022)		(0.142)	(0.061)	(0.063)
Education yrs. per capita (log)	-0.621***	-0.821***	-0.300***	0.300***	-0.766**	-0.800***	-1.005***
	(0.089)	(0.120)	(0.051)	(0.046)	(0.213)	(0.189)	(0.178)
HIV Prevalence (log)	0.033*	0.075*		0.033***	0.044	-0.154***	-0.102***
	(0.010)	(0.023)		(0.007)	(0.039)	(0.019)	(0.018)
Urbanicity (logit)	-0.027		-0.020	0.001	-0.109		-0.086*
	(0.017)		(0.010)	(0.006)	(0.061)		(0.028)
OOP as a share of THE (logit)	-0.014	0.025		-0.027*	-0.044	0.015	
	(0.013)	(0.021)		(0.008)	(0.038)	(0.019)	
GHES as a share of THE (logit)	0.019	-0.015		-0.004	0.049	-0.049*	-0.028
	(0.012)	(0.018)		(0.006)	(0.029)	(0.015)	(0.015)
Population over 65 (logit)	0.185***	0.145	0.072**	-0.001	0.600***	-0.042	0.126
	(0.034)	(0.066)	(0.019)	(0.021)	(0.096)	(0.065)	(0.063)
Observations	1,026	1,026	1,026	1,026	1,026	1,026	1,026
R^2	0.197	0.230	0.245	0.141	0.331	0.341	0.297
Adjusted R^2	0.126	0.162	0.180	0.068	0.272	0.282	0.235
Note:							*p<0.05; **p<0.01; ***p<0.001

Upper Middle Income Countries: Fixed Effects: Health Outputs

	Dependent variable:								
	HAQ Index	Health worker Density	Smoking Prevalence	UHC index	ANC4 coverage	ART Coverage	DTP3 coverage	Measles Vaccine Coverage	SBA coverage
PHC/THE	5.003***	-2.646**	-3.162	2.162**	12.521**	-18.220	45.795***	22.325*	-2.593
	(0.489)	(0.769)	(1.087)	(0.539)	(3.496)	(17.503)	(6.916)	(7.051)	(5.318)
THE per capita (log)	-0.008	0.131***	0.079**	-0.009	0.327***	0.223	0.235	0.415***	0.100
	(0.008)	(0.013)	(0.022)	(0.008)	(0.049)	(0.293)	(0.095)	(0.101)	(0.074)
LDI per capita (log)	0.172***	0.256***	0.010	0.095***		1.802**			1.205***
	(0.017)	(0.025)	(0.027)	(0.016)		(0.508)			(0.157)
Fertility rate (log)	-0.124***	-0.083	0.047	0.044				-0.201	-0.780***
	(0.015)	(0.031)	(0.031)	(0.019)				(0.326)	(0.153)
Hospital beds per 1000 (log)	0.175***	0.188***		-0.063	-0.293	-0.017	-0.269	-1.848***	1.361***
	(0.018)	(0.029)		(0.026)	(0.118)	(0.677)	(0.241)	(0.350)	(0.177)
Education yrs. per capita (log)		0.831***	0.270**	0.084		6.035***	-0.130	1.681	3.318***
		(0.057)	(0.071)	(0.039)		(1.288)	(0.491)	(0.611)	(0.299)
HIV Prevalence (log)	-0.013	0.003	-0.013	-0.028***	0.190***	1.203***	0.049		0.236***
	(0.005)	(0.007)	(0.011)	(0.006)	(0.036)	(0.151)	(0.073)		(0.038)
Urbanicity (logit)	0.054***	0.113***	0.154***	-0.012	0.539***	-1.578***	0.319	0.057	-0.309**
	(0.006)	(0.016)	(0.017)	(0.010)	(0.090)	(0.209)	(0.113)	(0.113)	(0.077)
OOP as a share of THE (logit)		0.022	0.062***	-0.002	0.027	0.686*	-0.085		-0.253***
		(0.012)	(0.012)	(0.005)	(0.041)	(0.210)	(0.081)		(0.049)
GHES as a share of THE (logit)	0.006	0.007	0.040**		0.167***	0.633*	-0.099	0.007	
	(0.005)	(0.012)	(0.011)		(0.037)	(0.185)	(0.069)	(0.066)	
Population over 65 (logit)	-0.125***	-0.135***	-0.207***	-0.194***	0.340	-2.844***	0.015	-0.422	
	(0.018)	(0.033)	(0.046)	(0.023)	(0.119)	(0.593)	(0.262)	(0.308)	
Observations	1,026	1,026	1,026	1,026	1,026	1,026	1,026	1,026	1,026
R^2	0.555	0.520	0.125	0.286	0.273	0.228	0.063	0.071	0.407
Adjusted R^2	0.516	0.477	0.048	0.224	0.211	0.160	-0.018	-0.009	0.355
Note:									*p<0.05; **p<0.01; ***p<0.001

E. Binary relationships between PHC expenditures and outcomes/outputs

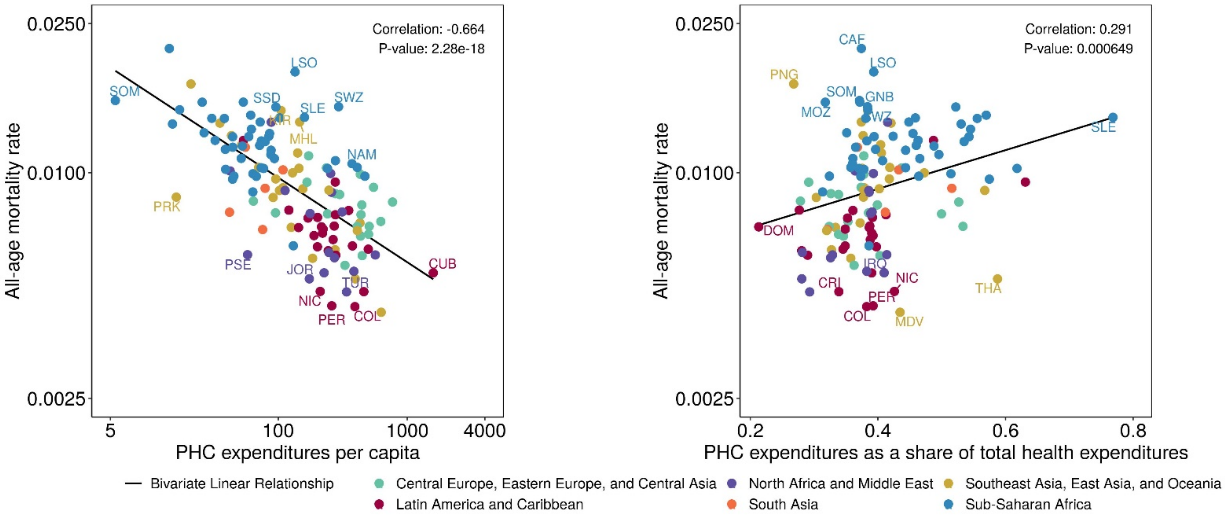

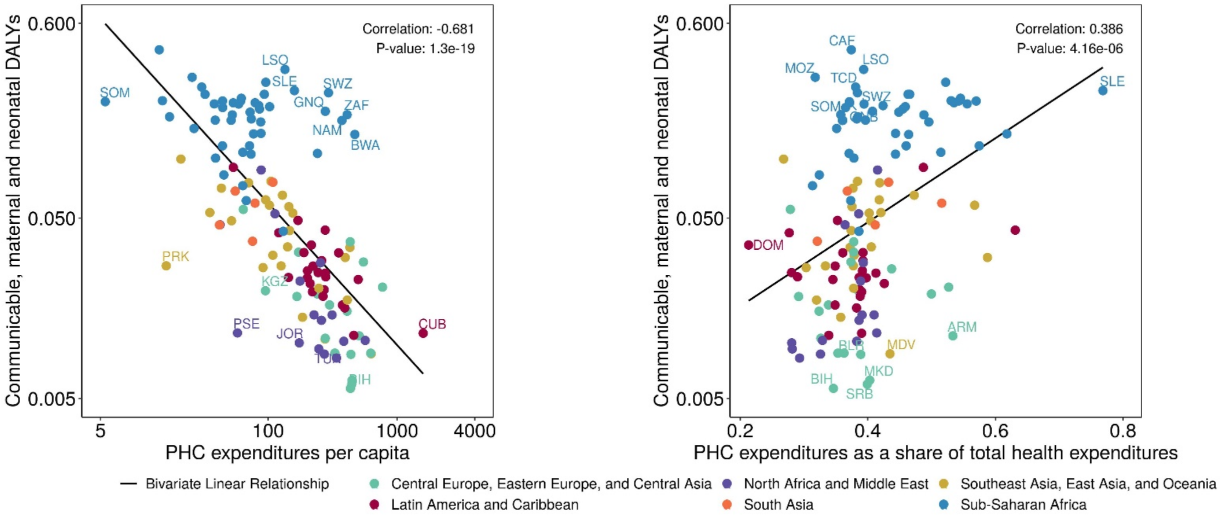

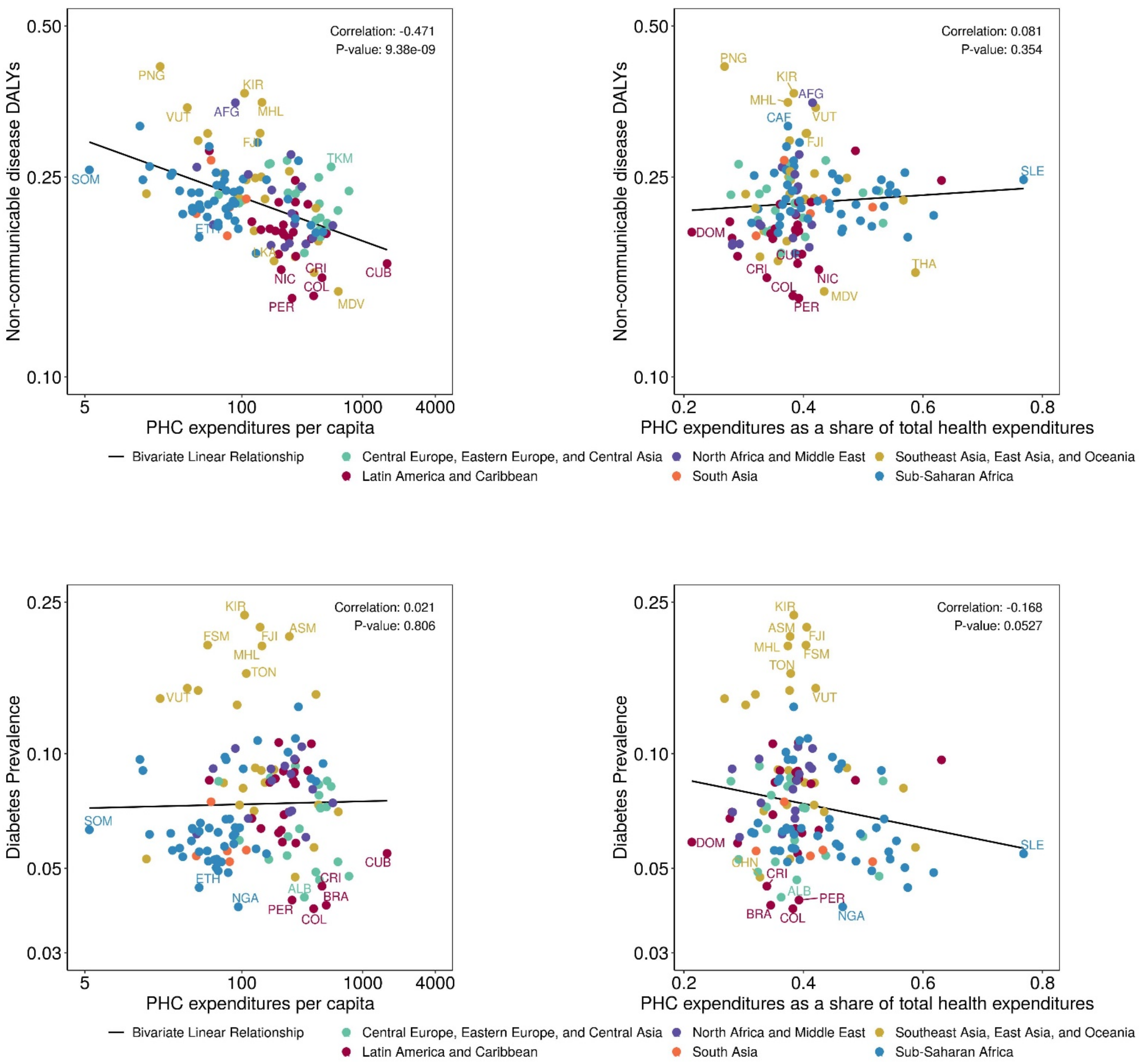
Correlation: -0.471
P-value: 9.38e-09
Non-communicable disease DALYs
PHC expenditures per capita
Correlation: 0.081
P-value: 0.354
PHC expenditures as a share of total health expenditures
Correlation: 0.021
P-value: 0.806
Diabetes Prevalence
Correlation: -0.168
P-value: 0.0527
Bivariate Linear Relationship
Central Europe, Eastern Europe, and Central Asia
Latin America and Caribbean
North Africa and Middle East
South Asia
Southeast Asia, East Asia, and Oceania
Sub-Saharan Africa

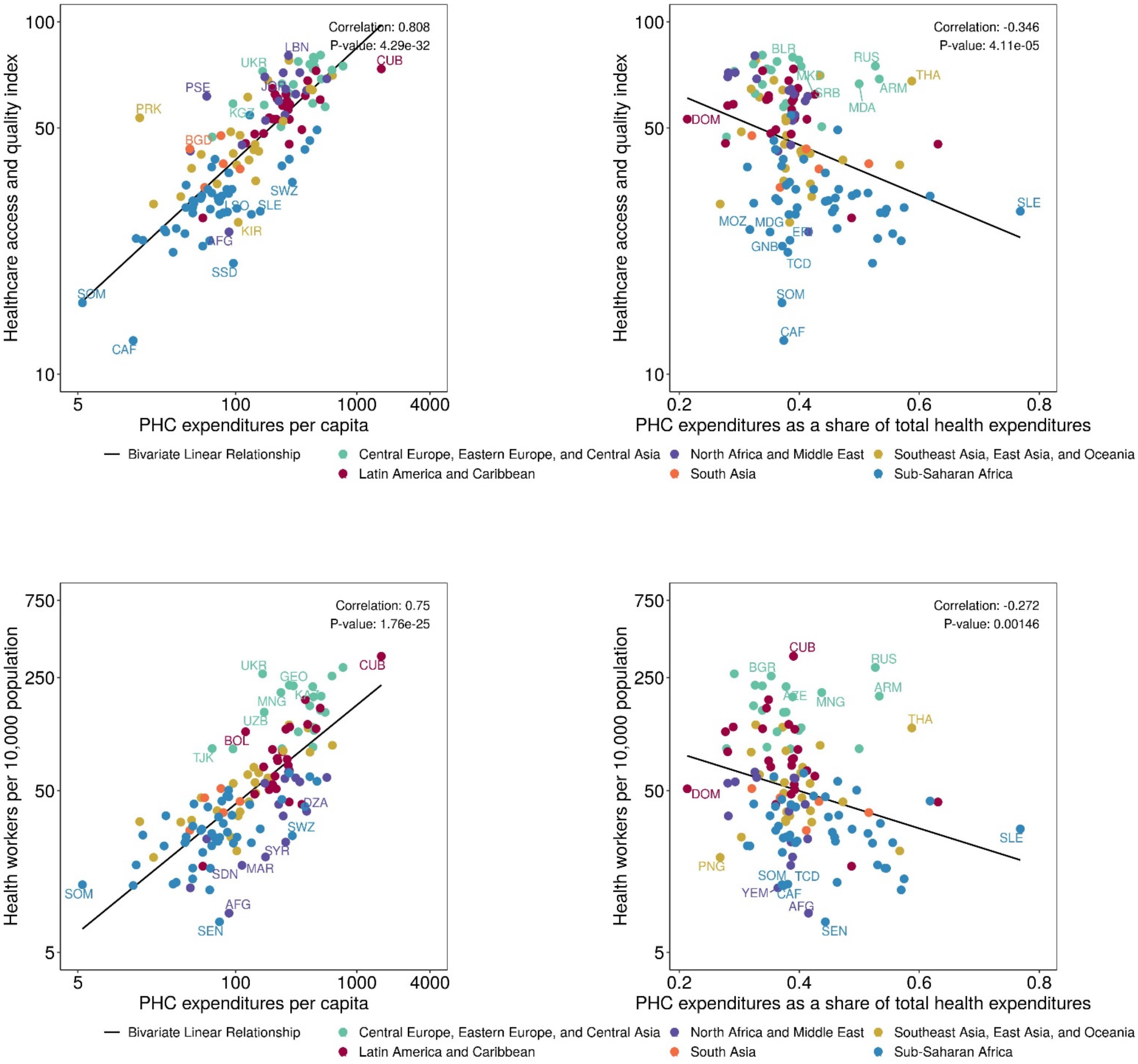
Correlation: 0.808
P-value: 4.29e-32
Healthcare access and quality index
PHC expenditures per capita
Correlation: -0.346
P-value: 4.11e-05
Healthcare access and quality index
PHC expenditures as a share of total health expenditures
Bivariate Linear Relationship
Central Europe, Eastern Europe, and Central Asia
North Africa and Middle East
Southeast Asia, East Asia, and Oceania
Latin America and Caribbean
South Asia
Sub-Saharan Africa
Correlation: 0.75
P-value: 1.76e-25
Health workers per 10,000 population
PHC expenditures per capita
Correlation: -0.272
P-value: 0.00146
Health workers per 10,000 population
PHC expenditures as a share of total health expenditures
Bivariate Linear Relationship
Central Europe, Eastern Europe, and Central Asia
North Africa and Middle East
Southeast Asia, East Asia, and Oceania
Latin America and Caribbean
South Asia
Sub-Saharan Africa

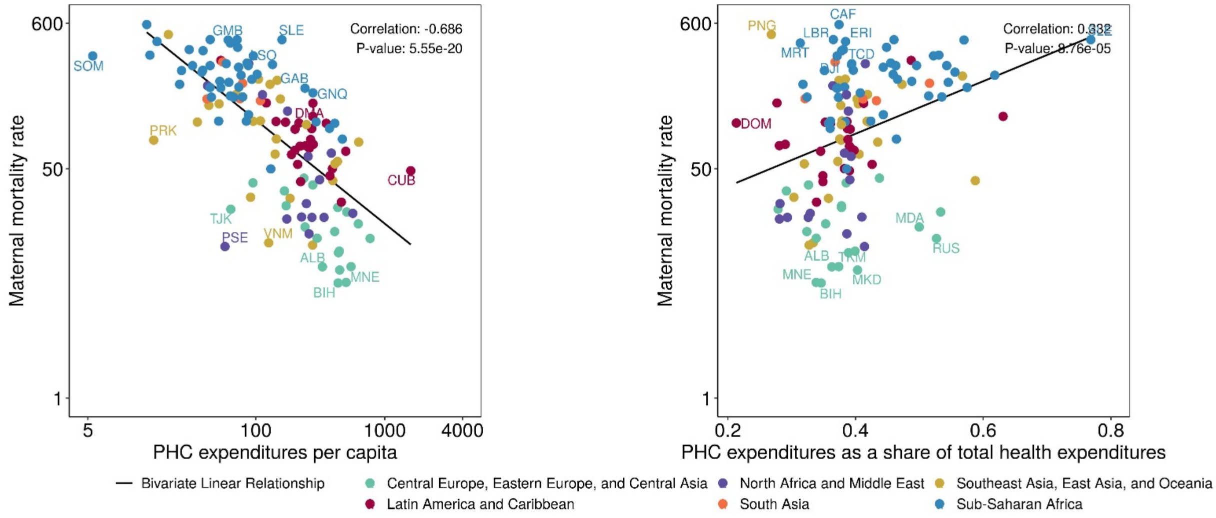
Correlation: -0.686
P-value: 5.55e-20
Maternal mortality rate
PHC expenditures per capita
Correlation: 0.332
P-value: 8.76e-05
PHC expenditures as a share of total health expenditures
Bivariate Linear Relationship
Central Europe, Eastern Europe, and Central Asia
Latin America and Caribbean
North Africa and Middle East
South Asia
Southeast Asia, East Asia, and Oceania
Sub-Saharan Africa

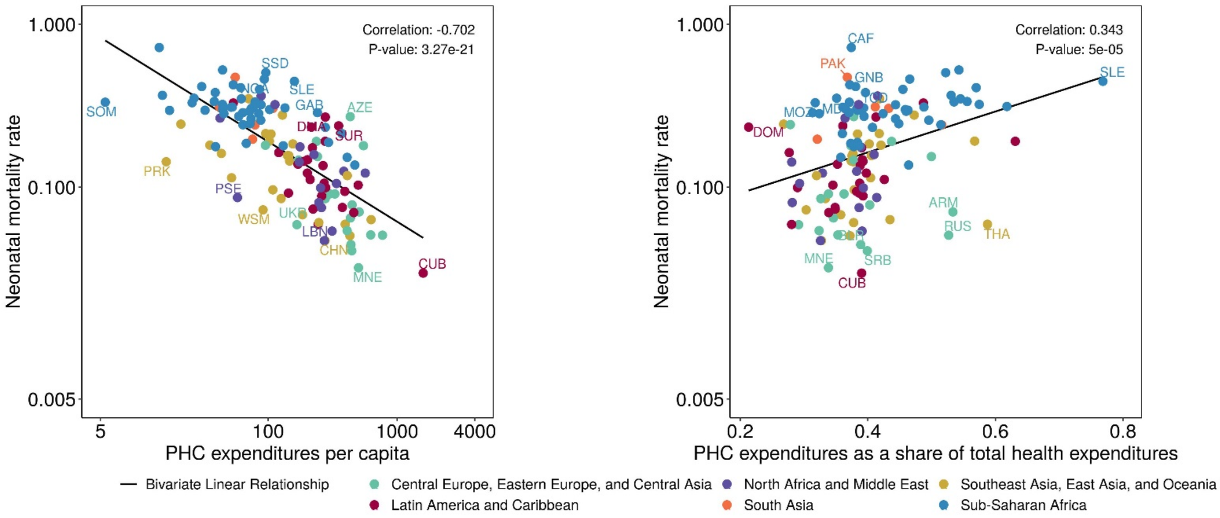
Correlation: -0.702
P-value: 3.27e-21
Neonatal mortality rate
PHC expenditures per capita
Correlation: 0.343
P-value: 5e-05
PHC expenditures as a share of total health expenditures
Bivariate Linear Relationship
Central Europe, Eastern Europe, and Central Asia
Latin America and Caribbean
North Africa and Middle East
South Asia
Southeast Asia, East Asia, and Oceania
Sub-Saharan Africa

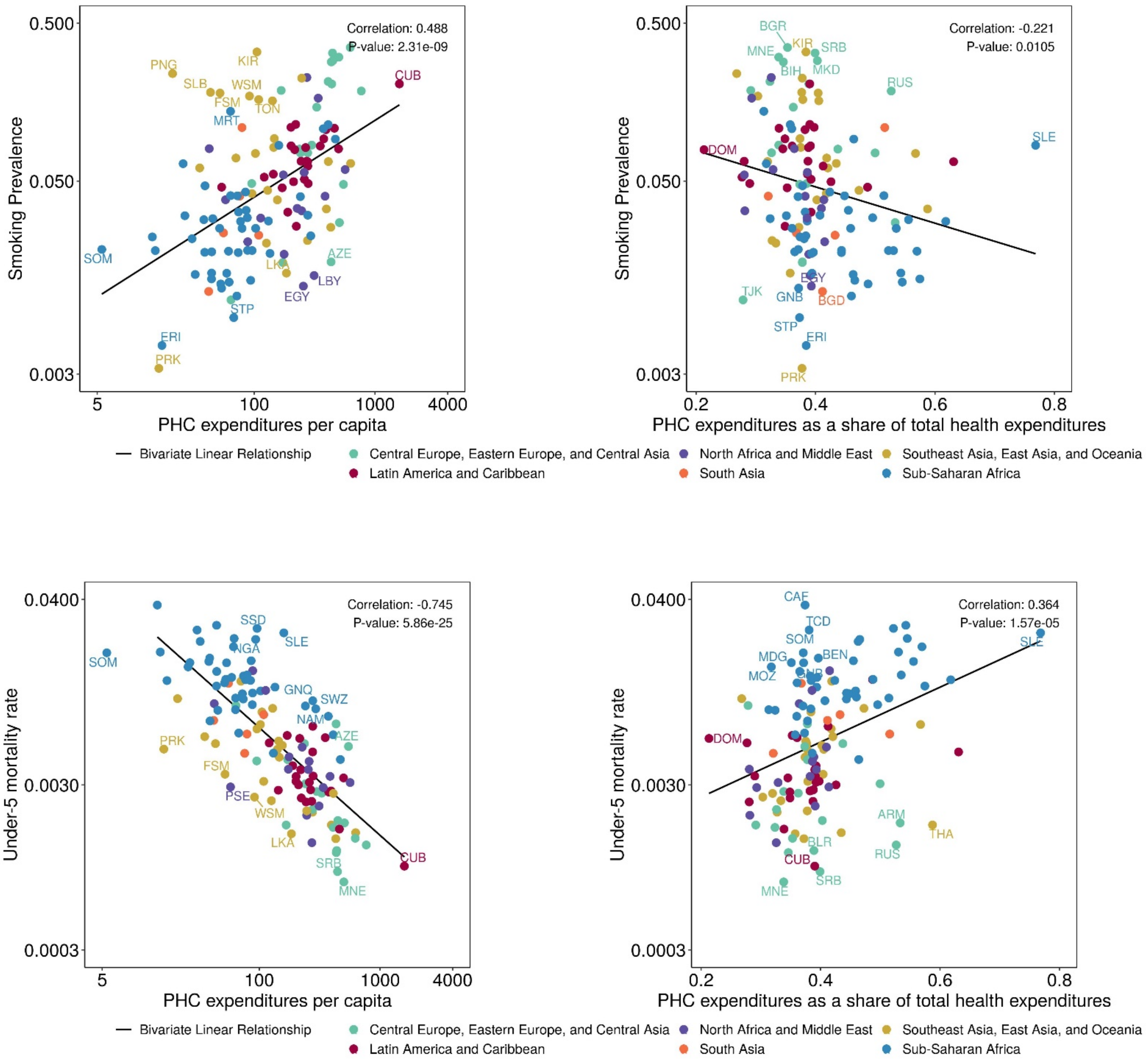
Correlation: 0.488
P-value: 2.31e-09
Smoking Prevalence
PHC expenditures per capita
Correlation: -0.221
P-value: 0.0105
PHC expenditures as a share of total health expenditures
Bivariate Linear Relationship
Central Europe, Eastern Europe, and Central Asia
North Africa and Middle East
Southeast Asia, East Asia, and Oceania
Latin America and Caribbean
South Asia
Sub-Saharan Africa
Correlation: -0.745
P-value: 5.86e-25
Under-5 mortality rate
PHC expenditures per capita
Correlation: 0.364
P-value: 1.57e-05
PHC expenditures as a share of total health expenditures
Bivariate Linear Relationship
Central Europe, Eastern Europe, and Central Asia
North Africa and Middle East
Southeast Asia, East Asia, and Oceania
Latin America and Caribbean
South Asia
Sub-Saharan Africa

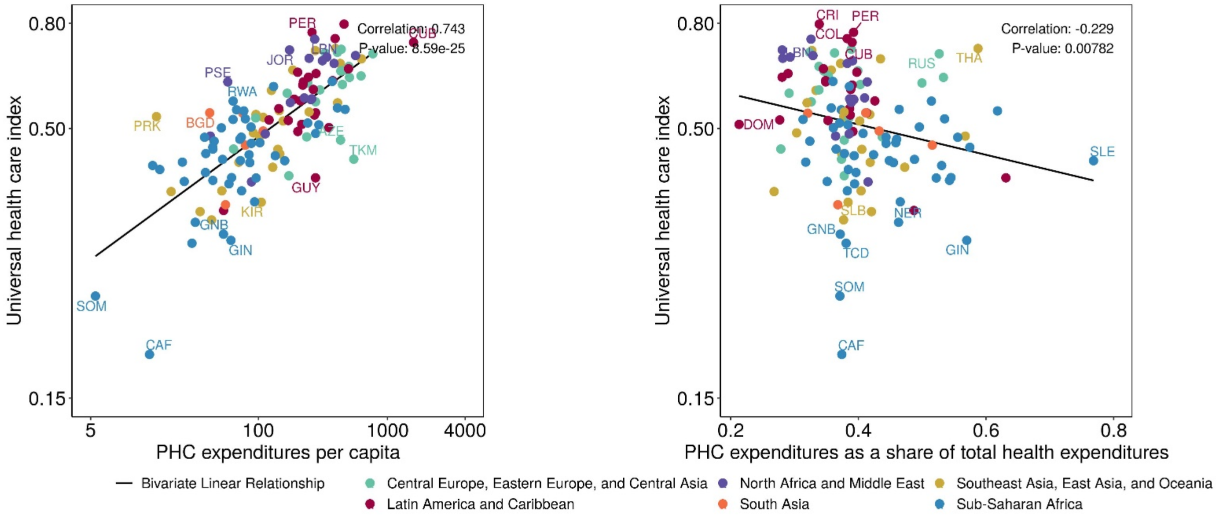
Correlation: 0.743
P-value: 8.59e-25
Universal health care index
PHC expenditures per capita
0.80
0.50
0.15
5
100
1000
4000
PER
CUB
JOR
PSE
RWA
PRK
BGD
TKM
GUY
KIR
GNB
GIN
SOM
CAF
Correlation: -0.229
P-value: 0.00782
PHC expenditures as a share of total health expenditures
0.2
0.4
0.6
0.8
CRI
PER
COL
CUB
RUS
THA
DOM
SLE
SLB
NER
GNB
TCD
GIN
SOM
CAF
Bivariate Linear Relationship
Central Europe, Eastern Europe, and Central Asia
Latin America and Caribbean
North Africa and Middle East
South Asia
Southeast Asia, East Asia, and Oceania
Sub-Saharan Africa

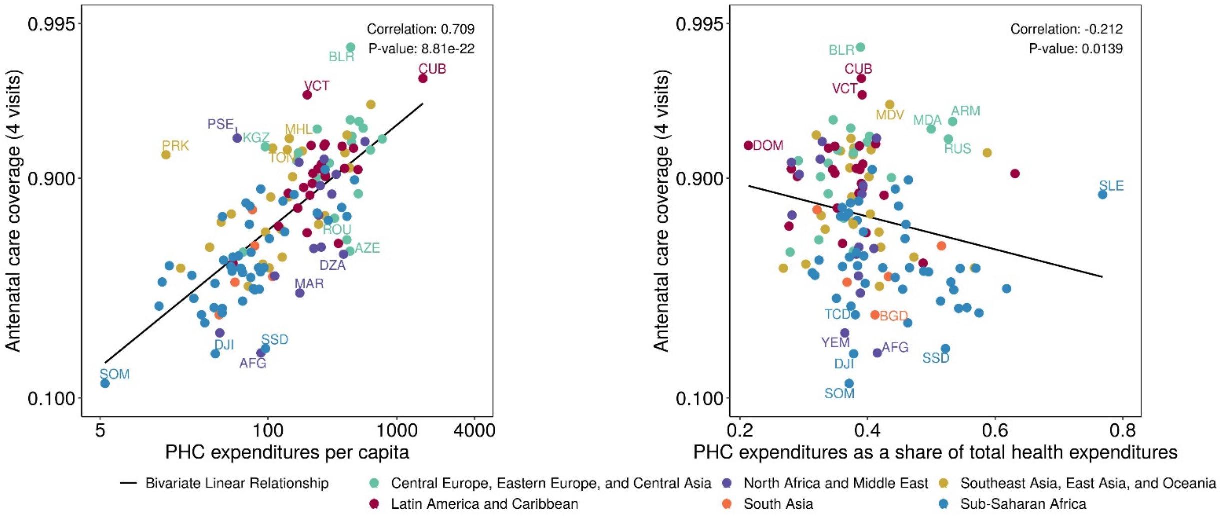
Correlation: 0.709
P-value: 8.81e-22
Antenatal care coverage (4 visits)
PHC expenditures per capita
0.995
0.900
0.100
5
100
1000
4000
BLR
CUB
VCT
PSE
KGZ
MHL
PRK
TON
ROU
AZE
DZA
MAR
DJI
SSD
AFG
SOM
Correlation: -0.212
P-value: 0.0139
PHC expenditures as a share of total health expenditures
0.2
0.4
0.6
0.8
BLR
CUB
VCT
MDV
MDA
ARM
DOM
RUS
SLE
TCD
BGD
YEM
AFG
DJI
SSD
SOM
Bivariate Linear Relationship
Central Europe, Eastern Europe, and Central Asia
Latin America and Caribbean
North Africa and Middle East
South Asia
Southeast Asia, East Asia, and Oceania
Sub-Saharan Africa

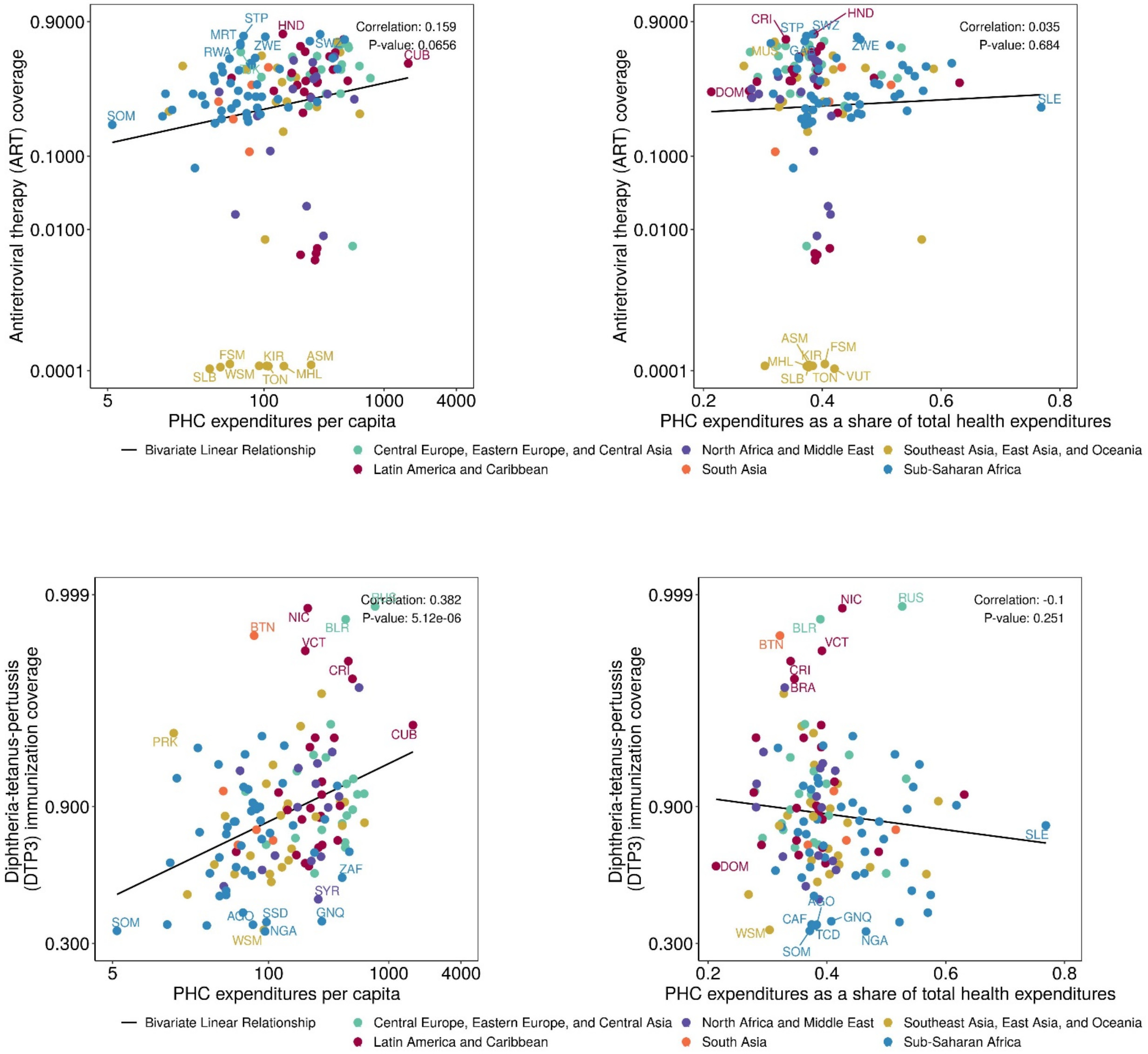
Correlation: 0.159
P-value: 0.0656
Antiretroviral therapy (ART) coverage
PHC expenditures per capita
Correlation: 0.035
P-value: 0.684
PHC expenditures as a share of total health expenditures
Bivariate Linear Relationship
Central Europe, Eastern Europe, and Central Asia
North Africa and Middle East
Southeast Asia, East Asia, and Oceania
Latin America and Caribbean
South Asia
Sub-Saharan Africa
Correlation: 0.382
P-value: 5.12e-06
Diphtheria-tetanus-pertussis (DTP3) immunization coverage
Correlation: -0.1
P-value: 0.251

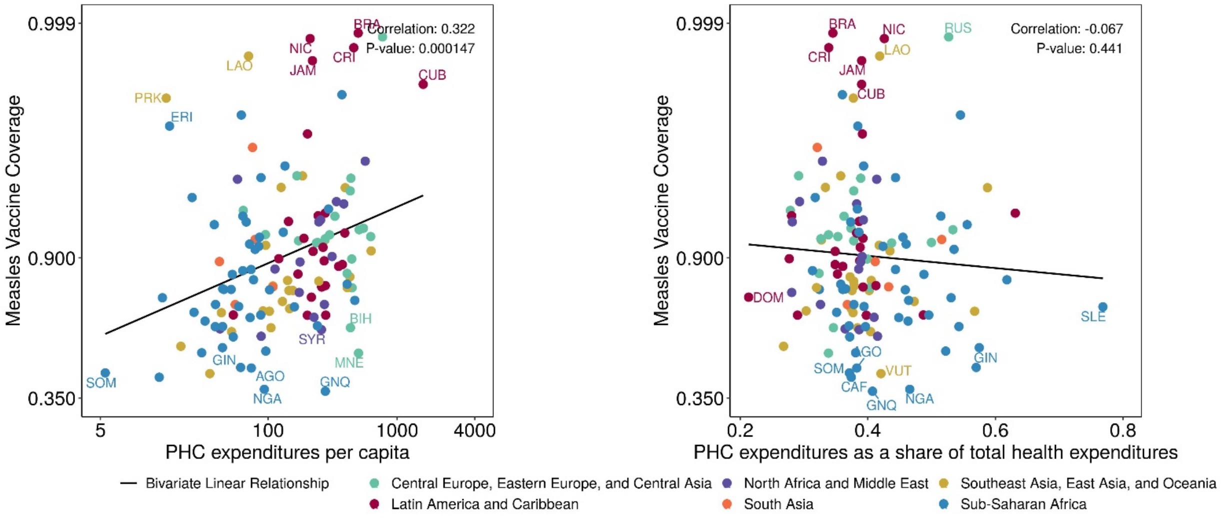
Correlation: 0.322
P-value: 0.000147
Measles Vaccine Coverage
PHC expenditures per capita
0.999
0.900
0.350
5
100
1000
4000
BRA
NIC
CRI
LAO
JAM
CUB
PRK
ERI
BIH
SYR
MNE
GIN
AGO
GNQ
SOM
NGA
Correlation: -0.067
P-value: 0.441
PHC expenditures as a share of total health expenditures
0.2
0.4
0.6
0.8
RUS
DOM
SLE
VUT
CAF
Bivariate Linear Relationship
Central Europe, Eastern Europe, and Central Asia
North Africa and Middle East
Southeast Asia, East Asia, and Oceania
Latin America and Caribbean
South Asia
Sub-Saharan Africa

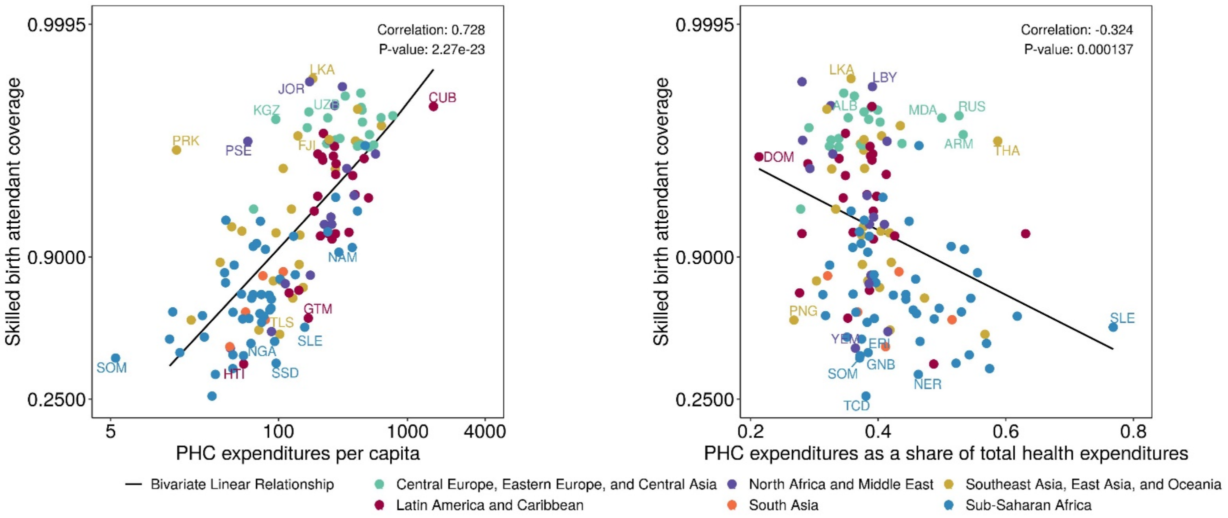
Correlation: 0.728
P-value: 2.27e-23
Skilled birth attendant coverage
PHC expenditures per capita
0.9995
0.9000
0.2500
5
100
1000
4000
LKA
JOR
CUB
KGZ
UZB
PRK
PSE
FJI
NAM
GTM
TLS
SLE
NGA
SOM
HTI
SSD
Correlation: -0.324
P-value: 0.000137
PHC expenditures as a share of total health expenditures
0.2
0.4
0.6
0.8
LBY
ALB
MDA
RUS
ARM
THA
DOM
PNG
YEM
ERI
GNB
NER
TCD
Bivariate Linear Relationship
Central Europe, Eastern Europe, and Central Asia
North Africa and Middle East
Southeast Asia, East Asia, and Oceania
Latin America and Caribbean
South Asia
Sub-Saharan Africa

F. Sensitivity analyses: 10-year difference, Mundlak, and ambulatory split model outputs

LMIC: 10-year Long Difference: Health Outcomes

	Dependent variable:						
	All-age mortality	Communicable DALYs	Non-communicable DALYs	Diabetes Prevalence	Maternal mortality	Neonatal mortality	Under-5 mortality
PHC/THE	1.317	1.091	0.063	-0.830	-10.629***	-3.607	-0.511
	(1.122)	(1.712)	(0.487)	(0.456)	(2.082)	(1.318)	(1.431)
THE per capita (log)	-0.020	-0.063	0.026*	-0.005	-0.027	-0.089**	-0.085*
	(0.017)	(0.025)	(0.008)	(0.007)	(0.034)	(0.023)	(0.028)
LDI per capita (log)	-0.158***	-0.297***	-0.124***	-0.015	-0.443***	-0.203***	-0.302***
	(0.027)	(0.049)	(0.012)	(0.009)	(0.051)	(0.035)	(0.035)
Fertility rate (log)	-0.035	-0.068	-0.036	-0.027	-0.389***	-0.343***	-0.179
	(0.034)	(0.055)	(0.017)	(0.016)	(0.078)	(0.052)	(0.062)
Hospital beds per 1000 (log)	-0.132**	0.071	-0.133***		-0.695***	-0.132	-0.073
	(0.036)	(0.062)	(0.021)		(0.105)	(0.069)	(0.056)
Education yrs. per capita (log)	-0.167**	-0.528***		0.180***	-0.206	-0.556***	-0.747***
	(0.047)	(0.079)		(0.022)	(0.115)	(0.092)	(0.098)
HIV Prevalence (log)	0.058***	0.079***	0.004	0.028***	-0.002	-0.099***	-0.045*
	(0.007)	(0.015)	(0.004)	(0.004)	(0.025)	(0.012)	(0.014)
Urbanicity (logit)	-0.091*	-0.156*	-0.033	-0.027*	-0.162	-0.080	-0.177**
	(0.029)	(0.046)	(0.014)	(0.008)	(0.065)	(0.034)	(0.048)
OOP as a share of THE (logit)		-0.006		-0.010	-0.038	-0.009	-0.004
		(0.017)		(0.005)	(0.023)	(0.012)	(0.015)
GHES as a share of THE (logit)	0.002	-0.028	0.006	0.007	0.001	-0.058***	-0.034
	(0.008)	(0.014)	(0.005)	(0.004)	(0.017)	(0.011)	(0.012)
Population over 65 (logit)	0.116***	0.162**	0.030	-0.055**	0.250***	-0.101	0.064
	(0.028)	(0.043)	(0.012)	(0.014)	(0.060)	(0.042)	(0.054)
Constant	-0.107***	-0.196***	-0.062***	0.035***	-0.154***	-0.113***	-0.159***
	(0.016)	(0.027)	(0.005)	(0.006)	(0.036)	(0.025)	(0.027)
Observations	1,080	1,080	1,080	1,080	1,080	1,073	1,080
R^2	0.165	0.230	0.156	0.126	0.238	0.300	0.226
Adjusted R^2	0.157	0.222	0.149	0.118	0.230	0.292	0.218
Note:							*p<0.05; **p<0.01; ***p<0.001

LMIC: 10-year Long Difference: Health Outputs

	Dependent variable:								
	HAQ Index	Health worker Density	Smoking Prevalence	UHC index	ANC4 coverage	ART Coverage	DTP3 coverage	Measles Vaccine Coverage	SBA coverage
PHC/THE	1.268	-1.042	-1.051	-0.334	12.756***	1.073	7.085	23.316**	-3.448
	(0.561)	(0.707)	(0.853)	(0.790)	(2.871)	(15.577)	(6.415)	(6.000)	(4.431)
THE per capita (log)	-0.007	0.139***	0.018	0.005	0.278***	1.227***	0.231	0.659***	0.181
	(0.011)	(0.013)	(0.016)	(0.013)	(0.051)	(0.250)	(0.094)	(0.102)	(0.071)
LDI per capita (log)	0.208***	0.279***	0.008	0.165***	0.430***	0.594		-0.517*	0.929***
	(0.012)	(0.022)	(0.022)	(0.017)	(0.068)	(0.379)		(0.156)	(0.110)
Fertility rate (log)	-0.081***	-0.318***	0.146***	0.075	-0.323	2.098**	-0.141	-0.866	-0.828***
	(0.020)	(0.032)	(0.036)	(0.026)	(0.111)	(0.551)	(0.297)	(0.322)	(0.137)
Hospital beds per 1000 (log)	0.070*	0.200***	0.076	-0.033	-0.161	0.486	-0.764	-1.417***	1.097***
	(0.021)	(0.037)	(0.043)	(0.025)	(0.146)	(0.632)	(0.278)	(0.331)	(0.204)
Education yrs. per capita (log)	0.466***	-0.058	0.028	0.209***	1.117***	6.991***	2.370***	2.137***	1.409***
	(0.033)	(0.055)	(0.057)	(0.040)	(0.160)	(0.867)	(0.347)	(0.409)	(0.188)
HIV Prevalence (log)	-0.003	0.027**	0.031**	-0.061***	0.155***	0.275	0.014		0.088
	(0.005)	(0.007)	(0.008)	(0.007)	(0.033)	(0.149)	(0.063)		(0.035)
Urbanicity (logit)	0.100***		0.102***	0.066	0.459***	-0.169	0.722***	0.460	-0.070
	(0.017)		(0.021)	(0.024)	(0.084)	(0.408)	(0.152)	(0.196)	(0.133)
OOP as a share of THE (logit)	-0.022*	-0.017	0.010	-0.005		0.110	-0.133	0.033	-0.121
	(0.007)	(0.009)	(0.010)	(0.009)		(0.179)	(0.054)	(0.061)	(0.048)
GHES as a share of THE (logit)	-0.009	0.0001		-0.011	0.044	-0.149		0.132	0.140**
	(0.006)	(0.009)		(0.009)	(0.024)	(0.163)		(0.054)	(0.039)
Population over 65 (logit)	-0.058***	-0.002	-0.077	-0.161***	0.529***	-3.653***	0.225	-0.262	0.002
	(0.013)	(0.026)	(0.034)	(0.021)	(0.080)	(0.469)	(0.235)	(0.205)	(0.108)
Constant	-0.013	0.156***	-0.066***	0.091***	-0.147	2.319***	-0.459**	-0.393	0.037
	(0.008)	(0.014)	(0.014)	(0.011)	(0.050)	(0.250)	(0.128)	(0.134)	(0.055)
Observations	1,080	1,080	1,080	1,080	1,080	1,080	1,080	1,080	1,080
R^2	0.495	0.424	0.055	0.302	0.251	0.206	0.078	0.090	0.276
Adjusted R^2	0.490	0.419	0.046	0.295	0.244	0.198	0.070	0.082	0.268
Note:									*p<0.05; **p<0.01; ***p<0.001

LMIC: Mundlak: Health Outcomes

	Dependent variable:						
	All-age mortality	Communicable DALYs	Non-communicable DALYs	Diabetes Prevalence	Maternal mortality	Neonatal mortality	Under-5 mortality
PHC/THE (Country Mean)	-0.248	0.934	-0.372	-0.349	0.241	0.136	-0.034
	(0.222)	(0.411)	(0.167)	(0.304)	(0.559)	(0.307)	(0.284)
PHC/THE (Dev)	1.463	1.590	0.022	-1.151	-11.310^{***}	-3.526^{*}	-0.380
	(0.798)	(1.273)	(0.426)	(0.409)	(1.615)	(1.115)	(1.168)
THE per capita (log)	-0.036^{*}	-0.112^{***}	0.002	-0.003	-0.095^{**}	-0.107^{***}	-0.113^{***}
	(0.011)	(0.019)	(0.006)	(0.006)	(0.024)	(0.016)	(0.019)
LDI per capita (log)	-0.161^{***}	-0.396^{***}	-0.140^{***}	0.011	-0.429^{***}	-0.244^{***}	-0.354^{***}
	(0.017)	(0.034)	(0.009)	(0.007)	(0.035)	(0.026)	(0.027)
Fertility rate (log)	0.040	0.047	0.093^{***}	-0.031	-0.315^{***}	-0.257^{***}	-0.003
	(0.025)	(0.044)	(0.011)	(0.012)	(0.063)	(0.040)	(0.048)
Hospital beds per 1000 (log)	0.127^{***}	0.210^{***}	0.017		-0.386^{***}	0.069	0.193^{***}
	(0.018)	(0.034)	(0.013)		(0.047)	(0.026)	(0.030)
Education yrs. per capita (log)	-0.416^{***}	-1.006^{***}		0.270^{***}	-0.610^{***}	-0.792^{***}	-1.075^{***}
	(0.022)	(0.042)		(0.012)	(0.045)	(0.037)	(0.039)
HIV Prevalence (log)	0.041^{***}	0.095^{***}	-0.007	0.035^{***}	0.017	-0.083^{***}	-0.031^{**}
	(0.005)	(0.011)	(0.003)	(0.004)	(0.015)	(0.008)	(0.009)
Urbanicity (logit)	-0.046^{***}	-0.051	-0.058^{***}	-0.030^{**}	-0.105^{**}	-0.013	-0.015
	(0.011)	(0.018)	(0.009)	(0.009)	(0.026)	(0.023)	(0.023)
OOP as a share of THE (logit)		-0.011		-0.012^{**}	-0.044	0.004	0.014
		(0.012)		(0.003)	(0.015)	(0.009)	(0.010)
GHES as a share of THE (logit)	-0.004	-0.035^{*}	0.011^{*}	0.007	0.001	-0.051^{***}	-0.032^{**}
	(0.006)	(0.010)	(0.004)	(0.003)	(0.012)	(0.008)	(0.009)
Population over 65 (logit)	0.001	-0.122^{**}	-0.011	-0.032	-0.013	-0.275^{***}	-0.192^{***}
	(0.019)	(0.033)	(0.009)	(0.011)	(0.046)	(0.028)	(0.037)
Constant	-2.102^{***}	2.587^{***}	-0.416^{**}	-2.944^{***}	10.216^{***}	1.267^{***}	-0.271
	(0.176)	(0.315)	(0.111)	(0.147)	(0.401)	(0.284)	(0.316)
Observations	2,430	2,430	2,430	2,430	2,430	2,414	2,430
R^2	0.587	0.763	0.508	0.566	0.462	0.722	0.769
Adjusted R^2	0.585	0.762	0.506	0.564	0.459	0.720	0.768

Note: *p<0.05; **p<0.01; ***p<0.001

LMIC: Mundlak: Health Outputs

	Dependent variable:								
	HAQ Index	Health worker Density	Smoking Prevalence	UHC index	ANC4 coverage	ART Coverage	DTP3 coverage	Measles Vaccine Coverage	SBA coverage
PHC/THE (Country Mean)	0.255	0.433	-2.700**	0.447	1.336	5.612	1.623	1.574	1.649
	(0.178)	(0.405)	(0.727)	(0.178)	(1.089)	(2.125)	(1.200)	(0.896)	(1.007)
PHC/THE (Dev)	1.969***	-1.760*	-0.598	-0.523	12.509***	18.335	9.136	20.557***	0.356
	(0.412)	(0.587)	(0.638)	(0.627)	(2.123)	(13.108)	(5.135)	(4.868)	(3.269)
THE per capita (log)	0.004	0.158***	0.018	0.015	0.218***	1.256***	0.170	0.470***	0.191***
	(0.007)	(0.011)	(0.012)	(0.009)	(0.034)	(0.194)	(0.063)	(0.067)	(0.047)
LDI per capita (log)	0.194***	0.319***	0.007	0.185***	0.435***	0.797*		-0.363**	0.768***
	(0.009)	(0.017)	(0.017)	(0.013)	(0.047)	(0.239)		(0.095)	(0.064)
Fertility rate (log)	-0.107***	-0.342***	0.146***	0.013	-0.309**	-0.447	-0.576	-1.148***	-0.778***
	(0.016)	(0.027)	(0.029)	(0.019)	(0.079)	(0.410)	(0.208)	(0.211)	(0.094)
Hospital beds per 1000 (log)	0.019	0.028	0.194***	-0.147***	0.010	-4.453***	-0.059	-0.132	0.445***
	(0.014)	(0.026)	(0.033)	(0.015)	(0.065)	(0.222)	(0.107)	(0.099)	(0.077)
Education yrs. per capita (log)	0.418***	0.395***	-0.127***	0.403***	0.838***	8.698***	0.819***	0.715***	1.444***
	(0.019)	(0.025)	(0.031)	(0.021)	(0.078)	(0.350)	(0.148)	(0.152)	(0.099)
HIV Prevalence (log)	-0.018***	0.057***	0.007	-0.038***	0.111***	0.644***	-0.082		0.072*
	(0.004)	(0.006)	(0.006)	(0.004)	(0.021)	(0.068)	(0.030)		(0.022)
Urbanicity (logit)	0.059***		0.033	0.032***	0.070	0.021	0.110	0.067	0.051
	(0.007)		(0.018)	(0.007)	(0.042)	(0.124)	(0.060)	(0.063)	(0.062)
OOP as a share of THE (logit)	-0.021***	-0.018	0.012	-0.024***		-0.539***	-0.158***	-0.043	-0.095*
	(0.005)	(0.007)	(0.008)	(0.006)		(0.129)	(0.037)	(0.041)	(0.029)
GHES as a share of THE (logit)	-0.011	-0.001		-0.012	0.038	-0.505***		0.106	0.134***
	(0.004)	(0.006)		(0.006)	(0.016)	(0.123)		(0.038)	(0.025)
Population over 65 (logit)	-0.037*	0.125***	-0.096**	-0.061***	0.467***	-0.414	-0.046	-0.334	0.107
	(0.011)	(0.020)	(0.027)	(0.015)	(0.054)	(0.308)	(0.153)	(0.145)	(0.072)
Constant	1.165***	0.378	-2.317***	-3.647***	-3.205***	-28.917***	-1.164	0.982	-6.908***
	(0.107)	(0.234)	(0.339)	(0.139)	(0.673)	(2.182)	(0.985)	(0.974)	(0.729)
Observations	2,430	2,430	2,430	2,430	2,430	2,430	2,430	2,430	2,430
R^2	0.836	0.864	0.164	0.678	0.613	0.612	0.151	0.161	0.688
Adjusted R^2	0.835	0.863	0.160	0.677	0.611	0.610	0.147	0.157	0.686
Note:									*p<0.05; **p<0.01; ***p<0.001

LMIC: Fixed Effects (PHC Ambulatory Split): Health Outcomes

	Dependent variable:						
	All-age mortality	Communicable DALYs	Non-communicable DALYs	Diabetes Prevalence	Maternal mortality	Neonatal mortality	Under-5 mortality
Ambulatory PHC/THE	2.377	-2.211	2.148	-0.150	-13.111**	8.834**	7.479***
	(1.652)	(2.429)	(0.989)	(0.877)	(3.512)	(2.421)	(1.733)
Non-Ambulatory PHC/THE	0.515	2.381	-0.815	-1.144*	-9.097***	-8.548***	-3.406
	(1.063)	(1.638)	(0.431)	(0.382)	(1.856)	(1.329)	(1.590)
THE per capita (log)	-0.024	-0.054	0.016	-0.010	-0.018	-0.119***	-0.101***
	(0.013)	(0.020)	(0.006)	(0.005)	(0.026)	(0.017)	(0.022)
LDI per capita (log)	-0.141***	-0.319***	-0.107***	-0.005	-0.462***	-0.129***	-0.253***
	(0.020)	(0.038)	(0.010)	(0.008)	(0.044)	(0.030)	(0.031)
Fertility rate (log)	-0.030	-0.065	-0.026	-0.030	-0.402***	-0.327***	-0.146
	(0.026)	(0.044)	(0.013)	(0.013)	(0.064)	(0.041)	(0.050)
Hospital beds per 1000 (log)	-0.124***	0.074	-0.124***		-0.703***	-0.087	-0.036
	(0.027)	(0.052)	(0.015)		(0.086)	(0.050)	(0.046)
Education yrs. per capita (log)	-0.198***	-0.558***		0.166***	-0.231	-0.528***	-0.738***
	(0.036)	(0.064)		(0.020)	(0.094)	(0.071)	(0.079)
HIV Prevalence (log)	0.054***	0.065***	0.007	0.028***	-0.006	-0.093***	-0.049***
	(0.006)	(0.013)	(0.003)	(0.004)	(0.021)	(0.010)	(0.011)
Urbanicity (logit)	-0.085**	-0.154***	-0.033	-0.025*	-0.137	-0.087*	-0.181***
	(0.023)	(0.037)	(0.012)	(0.008)	(0.052)	(0.028)	(0.041)
OOP as a share of THE (logit)		-0.006		-0.009	-0.037	0.004	0.005
		(0.012)		(0.003)	(0.016)	(0.009)	(0.011)
GHES as a share of THE (logit)	0.002	-0.030	0.009	0.009*	-0.00002	-0.026*	-0.015
	(0.006)	(0.011)	(0.003)	(0.003)	(0.012)	(0.008)	(0.009)
Population over 65 (logit)	0.109***	0.151***	0.023	-0.051***	0.219***	-0.141***	0.041
	(0.023)	(0.035)	(0.009)	(0.012)	(0.048)	(0.033)	(0.045)
Observations	2,430	2,430	2,430	2,430	2,430	2,414	2,430
R^2	0.152	0.226	0.147	0.110	0.219	0.313	0.212
Adjusted R^2	0.092	0.170	0.087	0.047	0.162	0.263	0.155

Note: *p<0.05; **p<0.01; ***p<0.001

LMIC: Fixed Effects (PHC Ambulatory Split): Health Outputs

	Dependent variable:								
	HAQ Index	Health worker Density	Smoking Prevalence	UHC index	ANC4 coverage	ART Coverage	DTP3 coverage	Measles Vaccine Coverage	SBA coverage
Ambulatory PHC/THE	-1.096	-7.076***	0.225	-2.330	11.984	-7.070	60.916***	32.917**	-13.746
	(0.569)	(1.127)	(0.961)	(0.882)	(4.308)	(21.325)	(8.384)	(8.771)	(8.587)
Non-Ambulatory PHC/THE	2.327**	1.493	-1.398	0.651	12.803***	-11.699	-1.319	16.131	2.058
	(0.600)	(0.720)	(0.800)	(0.786)	(2.321)	(12.064)	(4.981)	(6.160)	(3.020)
THE per capita (log)	-0.002	0.150***	0.004	0.009	0.234***	0.540	0.169	0.522***	0.192**
	(0.008)	(0.010)	(0.012)	(0.011)	(0.037)	(0.184)	(0.067)	(0.082)	(0.050)
LDI per capita (log)	0.199***	0.248***	0.026	0.151***	0.470***	1.144**		-0.345	0.865***
	(0.011)	(0.017)	(0.020)	(0.016)	(0.057)	(0.311)		(0.131)	(0.076)
Fertility rate (log)	-0.084***	-0.336***	0.137***	0.073**	-0.356***	1.819***	-0.337	-0.890**	-0.795***
	(0.015)	(0.025)	(0.027)	(0.020)	(0.082)	(0.409)	(0.241)	(0.256)	(0.098)
Hospital beds per 1000 (log)	0.070**	0.193***	0.092	-0.032	-0.125	0.695	-0.736**	-1.339***	1.007***
	(0.018)	(0.028)	(0.035)	(0.021)	(0.106)	(0.516)	(0.209)	(0.262)	(0.141)
Education yrs. per capita (log)	0.465***	-0.080	-0.011	0.199***	1.084***	5.923***	2.169***	2.272***	1.337***
	(0.028)	(0.043)	(0.045)	(0.033)	(0.123)	(0.672)	(0.272)	(0.349)	(0.141)
HIV Prevalence (log)	-0.011	0.019*	0.025**	-0.063***	0.146***	0.128	0.045		0.067
	(0.005)	(0.006)	(0.007)	(0.006)	(0.027)	(0.115)	(0.048)		(0.028)
Urbanicity (logit)	0.105***		0.096***	0.067**	0.421***	-0.565	0.702***	0.365	-0.039
	(0.015)		(0.018)	(0.019)	(0.069)	(0.380)	(0.107)	(0.141)	(0.096)
OOP as a share of THE (logit)	-0.019**	-0.015	0.011	-0.008		0.355	-0.128*	0.072	-0.094*
	(0.005)	(0.006)	(0.007)	(0.006)		(0.127)	(0.039)	(0.044)	(0.030)
GHES as a share of THE (logit)	-0.008	-0.011		-0.012	0.041	0.263		0.192***	0.127***
	(0.004)	(0.006)		(0.006)	(0.017)	(0.111)		(0.041)	(0.023)
Population over 65 (logit)	-0.056***	0.015	-0.083*	-0.158***	0.549***	-2.903***	0.077	-0.130	0.077
	(0.011)	(0.021)	(0.027)	(0.017)	(0.063)	(0.388)	(0.179)	(0.165)	(0.081)
Observations	2,430	2,430	2,430	2,430	2,430	2,430	2,430	2,430	2,430
R^2	0.507	0.440	0.049	0.283	0.252	0.138	0.091	0.083	0.266
Adjusted R^2	0.471	0.400	-0.019	0.232	0.198	0.076	0.026	0.018	0.213

Note: *p<0.05; **p<0.01; ***p<0.001

References

1 MIDAS®. https://www.iqvia.com/solutions/commercialization/brand-strategy-and-management/market-measurement/midas (accessed April 30, 2020).

2 WHO | WHO Model Lists of Essential Medicines. WHO. http://www.who.int/medicines/publications/essentialmedicines/en/ (accessed April 30, 2020).

3 Gakidou E, Afshin A, Abajobir AA, *et al.* Global, regional, and national comparative risk assessment of 84 behavioural, environmental and occupational, and metabolic risks or clusters of risks, 1990–2016: a systematic analysis for the Global Burden of Disease Study 2016. *The Lancet* 2017; **390**: 1345–422.

4 Global Burden of Disease Collaborative Network. Global Burden of Disease Study 2017 (GBD 2017) Covariates 1980-2017. Seattle, United States: Institute for Health Metrics and Evaluation (IHME), 2018. http://ghdx.healthdata.org/record/ihme-data/gbd-2017-covariates-1980-2017 (accessed June 27, 2020).

5 Health Care Utilisation : Hospital discharges by diagnostic categories. OECD Stat. 2020. https://stats.oecd.org/index.aspx?queryid=30166 (accessed April 23, 2020).

6 Caesarean sections (indicator). OECD Data. 2020. http://data.oecd.org/healthcare/caesarean-sections.htm (accessed April 23, 2020).

7 Healthcare Cost and Utilization Project. HCUPnet. https://hcupnet.ahrq.gov/#setup (accessed April 23, 2020).

8 Patient Cost Estimator | CIHI. Canadian Institute for Health Information. 2019; published online Oct 31. https://www.cihi.ca/en/patient-cost-estimator (accessed April 23, 2020).

9 The DHS Program - Survey Search. https://dhsprogram.com/What-We-Do/survey-search.cfm?pgType=main&SrvyTp=type (accessed Feb 2, 2018).

10 Multiple Indicators Cluster Surveys - UNICEF MICS. https://mics.unicef.org/surveys (accessed June 30, 2020).

11 International comparisons of health care prices from the 2017 iFHP survey. HCCI. https://healthcostinstitute.org/hcci-research/international-comparisons-of-health-care-prices-2017-ifhp-survey (accessed April 23, 2020).

12 Abate KH, Abay SM, Abbafati C, *et al.* Global, regional, and national age-sex-specific mortality and life expectancy, 1950–2017: a systematic analysis for the Global Burden of Disease Study 2017. *The Lancet* 2018; **392**: 1684–735.

13 Entringer AP, Pinto MFT, Gomes MA de SM. Costs analysis of hospital care for vaginal delivery and elective caesarean section for usual risk pregnant women in the Brazilian Unified National Health System. *Ciênc saúde coletiva* 2019; **24**: 1527–36.

14 Entringer AP, Pinto M, Dias MAB, *et al.* Análise de custo-efetividade do parto vaginal espontâneo e da cesariana eletiva para gestantes de risco habitual no Sistema Único de Saúde. *Cadernos de Saúde Pública* 2018; **34**. DOI:10.1590/0102-311x00022517.

15 Gonzalez-Perez GJ, Vega-Lopez MG, Cabrera-Pivaral C, Muñoz A, Valle A. Caesarean sections in Mexico: are there too many? *Health Policy Plan* 2001; **16**: 62–7.

16 Bellanger MM, Quentin W, Tan SS. Childbirth and Diagnosis Related Groups (DRGs): patient classification and hospital reimbursement in 11 European countries. *European Journal of Obstetrics & Gynecology and Reproductive Biology* 2013; **168**: 12–9.

17 Iyengar SD, Iyengar K, Suhalka V, Agarwal K. Comparison of Domiciliary and Institutional Delivery-care Practices in Rural Rajasthan, India. *J Health Popul Nutr* 2009; **27**: 303–12.

18 Borghi J, Ensor T, Neupane BD, Tiwari S. Coping with the Burden of the Costs of Maternal Health. 2004; : 138.

19 Kim SJ, Kim SJ, Han K-T, Park E-C. Medical costs, Cesarean delivery rates, and length of stay in specialty hospitals vs. non-specialty hospitals in South Korea. *PLoS One* 2017; **12**. DOI:10.1371/journal.pone.0188612.

20 Gibbons L, Belizán JM, Lauer JA, Betrán AP, Merialdi M, Althabe F. The Global Numbers and Costs of Additionally Needed and Unnecessary Caesarean Sections Performed per Year: Overuse as a Barrier to Universal Coverage. *World Health Report* 2010; **30**: 1–31.

21 Witter S, Dieng T, Mbengue D, Moreira I, De Brouwere V. The national free delivery and caesarean policy in Senegal: evaluating process and outcomes. *Health Policy Plan* 2010; **25**: 384–92.

22 Ridde V, Richard F, Bicaba A, Queuille L, Conombo G. The national subsidy for deliveries and emergency obstetric care in Burkina Faso. *Health Policy Plan* 2011; **26**: ii30–40.

23 Moses MW, Pedroza P, Baral R, *et al.* Funding and services needed to achieve universal health coverage: applications of global, regional, and national estimates of utilisation of outpatient visits and inpatient admissions from 1990 to 2016, and unit costs from 1995 to 2016. *Lancet Public Health* 2018; **4**: e49–73.

24 Schneider M, Chang AY, Chapin A, *et al.* Modeling health expenditures by services and providers for 195 countries, 1995 to 2017. *Publication submitted for publication*.
